# Enhancing the selectivity of prolinamide organocatalysts using the mechanical bond in [2]rotaxanes†

**DOI:** 10.1039/d0sc00444h

**Published:** 2020-03-11

**Authors:** María Calles, Julio Puigcerver, Diego A. Alonso, Mateo Alajarin, Alberto Martinez-Cuezva, Jose Berna

**Affiliations:** Departamento de Química Orgánica, Facultad de Química, Regional Campus of International Excellence “Campus Mare Nostrum”, Universidad de Murcia E-30100 Murcia Spain ppberna@um.es amcuezva@um.es; Departamento de Química Orgánica, Facultad de Ciencias, Universidad de Alicante E-03080 Alicante Spain

## Abstract

The synthesis of a pair of switchable interlocked prolinamides and their use as organocatalysts in three different enamine-activated processes are reported. A diacylaminopyridine moiety was incorporated into the thread for directing [2]rotaxane formation further allowing the association of complementary small molecules. The rotaxane-based systems were tested as organocatalysts in asymmetric enamine-mediated processes, revealing a significantly improved catalytic ability if compared with the non-interlocked thread. The presence of an electron-withdrawing nitro group at the macrocycle helps to achieve high conversions and enantioselectivities. These systems are able to interact with *N*-hexylthymine as a cofactor to form supramolecular catalysts displaying a divergent catalytic behaviour. The presence or absence of the cofactor controls the chemoselectivity in competitive reactions.

## Abstract 2

The mechanical bonding and the cofactor assembly in interlocked prolinamide-based organocatalysts upgrade enamine-type transformations by increasing their yields and enantio- and chemo-selectivities.

## Graphical abstract

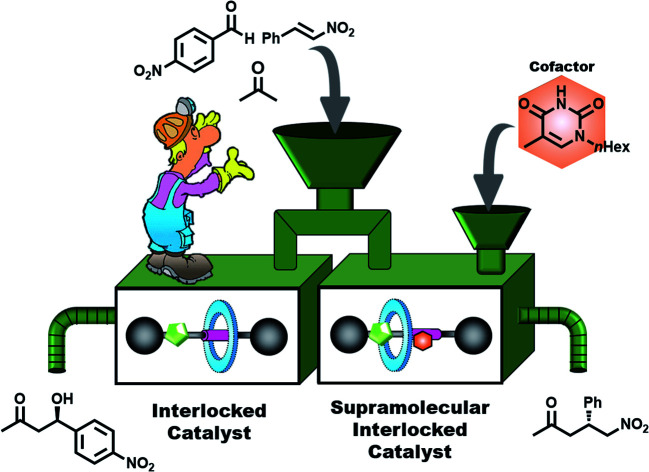

## 1. Introduction

Asymmetric organocatalysis has become a powerful tool for the synthesis of sophisticated molecules from easily available starting materials.^1^ In this area, (*S*)-proline was found to be a promising catalyst for asymmetric aldol transformations,^2,3^ and consequently the development of catalytic scaffolds bearing this privileged moiety is still a hot topic.

During the last few decades the interest of the scientific community in the synthesis and study of mechanically interlocked molecules (MIMs) has undergone a huge evolution.^4^ Within the range of applications of rotaxanes, the most frequently employed MIMs, those related to the study of their chemical reactivity, for example their use as organocatalysts^5^ or ligands in metal catalysed transformations,^6^ are lately capturing the attention of chemists. In this regard, switchable rotaxane-based catalysts, which are able to change their catalytic activity (ON/OFF, enantio- or diastereo-selectivity alterations and diverse activation modes), are of high interest.^7^ In general, the macrocycle inhibits catalysis when located over the catalytically active site.^8^ As a result, non-interlocked threads are more reactive than the corresponding interlocked systems, although displaying poorer selectivities. In contrast, the more rigid and confined interlocked catalysts^9^ oftentimes afford higher diastereo- or enantio-selectivities.^10^ In this line, we have demonstrated that the location of a polyamide ring close to a pyrrolidine active site of an interlocked catalyst switches the enantioselective course of a process, generating both possible enantiomers of the final products *via* an enantiodivergent approach.^11^ Recently, Leigh and coworkers reported controlled dynamic switching allowing the enantioselectivity of a rotaxane-catalyzed reaction to be reversed, thus highlighting the importance of the position of the ring with regard to the catalytically active site.^12^

**Fig. 1:**
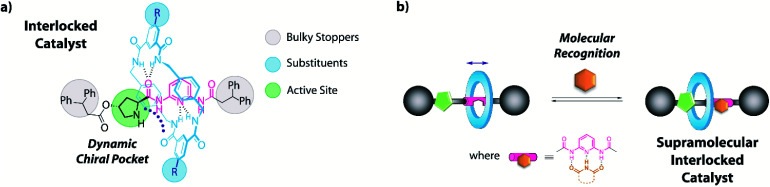
Design of (a) an interlocked DAP-based organocatalyst and (b) the formation of its hydrogen-bonding supramolecular complex.

Keeping in mind the potential of mechanically interlocked architectures for acting as catalysts,^13^ herein we have designed a series of chiral prolinamide-based rotaxanes bearing a diacylaminopyridine (DAP) moiety as the template,^14^ ready to be used as organocatalysts (Fig. 1).^15^ The selection of the DAP functionality as a binding site might enable these systems to modulate their activity by interaction with complementary molecules, such as *N*-hexylthymine acting as a supramolecular co-catalyst.^16,17^ Furthermore, we postulated that the proximity of the macrocycle to the catalytically active site (pyrrolidine core, Fig. 1, green circle) and its restricted translational motion between the bulky groups (Fig. 1, grey circle) could influence the course of the studied processes with unforeseeable trends. The position of the ring near the active site would generate a restricted dynamic chiral pocket leading to an increase of the selectivity of the catalytic process. In this context, the effect of the strength of the interaction between the interlocked components on the catalytic behaviour has been also studied by varying the acidity the NH of the isophthalamide units through the incorporation of different substituents^18^ on the macrocycle (Fig. 1, blue circle).

## 2. Results and discussion

### 2.1 Synthesis of mechanically interlocked DAP-based prolinamides

**Scheme 1:**
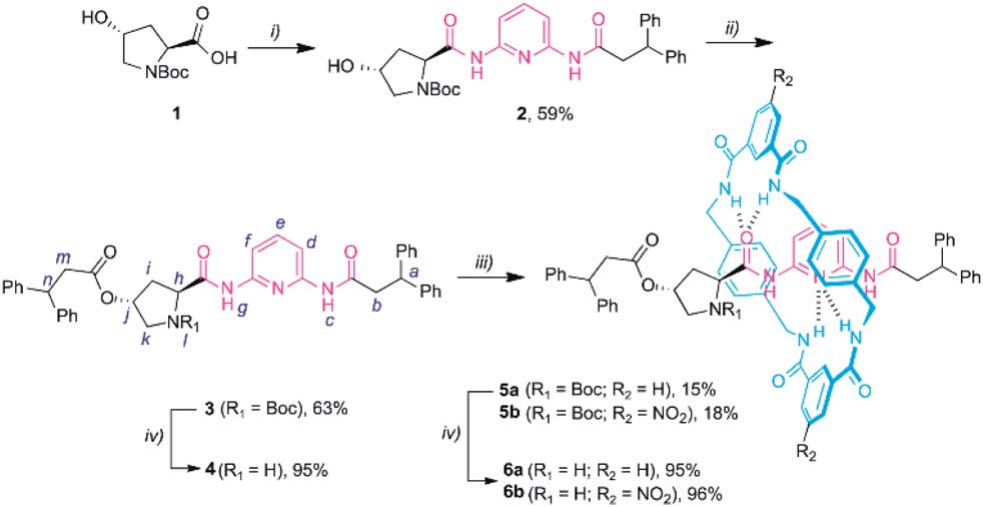
Synthesis of DAP-based non-interlocked and interlocked catalysts **4** and **6a-b**. Reaction conditions: (i) ethyl chloroformate, Et_3_N, THF, 0 °C; then addition of *N*-(6-aminopyridin-2-yl)-3,3-diphenylpropanamide **S1**; 25 °C, overnight; then reflux for 3 h; (ii) 3,3-diphenylpropanoyl chloride, Et_3_N, CH_2_Cl_2_, 25 °C, overnight; (iii) *p*-xylylenediamine, isophthaloyl dichloride, Et_3_N, CHCl_3_, 25 °C, 4 h; (iv) TFA, CHCl_3_, overnight. Experimental procedures can be found in the ESI.†

We started the synthesis from the commercially available Boc protected *trans*-4-hydroxy-l-proline **1** (Scheme 1). The amidation reaction between **1** and *N*-(6-aminopyridin-2-yl)-3,3-diphenylpropanamide (**S1**),^14*a*^ in the presence of ethyl chloroformate and Et_3_N, yielded compound **2** in 59% yield. Then, the esterification of **2** with 3,3-diphenylpropanoyl chloride provided the protected thread **3** in moderate yield. The corresponding Leigh-type [2]rotaxanes **5a,b** were readily obtained by carrying out a five-component reaction with *p*-xylylenediamine and a suitable isophthaloyl chloride (R_2_ = H or NO_2_) (see the ESI† for further details).^19^ Boc deprotection of the resulting prolinamides afforded the thread **4** and the rotaxanes **6a,b** (in only 3 and 4 synthetic steps, respectively), which can be directly tested as organocatalysts.

We investigated the ring location in the thread in rotaxane **6a**. The comparison of the ^1^H NMR spectra of thread **4** and rotaxane **6a** recorded in CDCl_3_ showed that the signals of the pyridine ring (H_d_, H_e_ and H_f_, see lettering in Scheme 1) are shifted to a higher field for **6a** as a result of binding with the benzylic amide macrocycle. However, the magnitude of this shifting is slightly smaller (see the ESI, Table S1†) than that in other DAP-based rotaxanes^14^ pointing out that the ring could be also interacting with other hydrogen bonding motifs of the thread. Indeed, the proximity of the five-membered ring to the encircled DAP unit of **6a** causes concomitant shifting of the ^1^H NMR signals of the pyrrolidine core indicating that the macrocycle also remains close to the active site, structurally defining a dynamic chiral pocket.

**Fig. 2:**
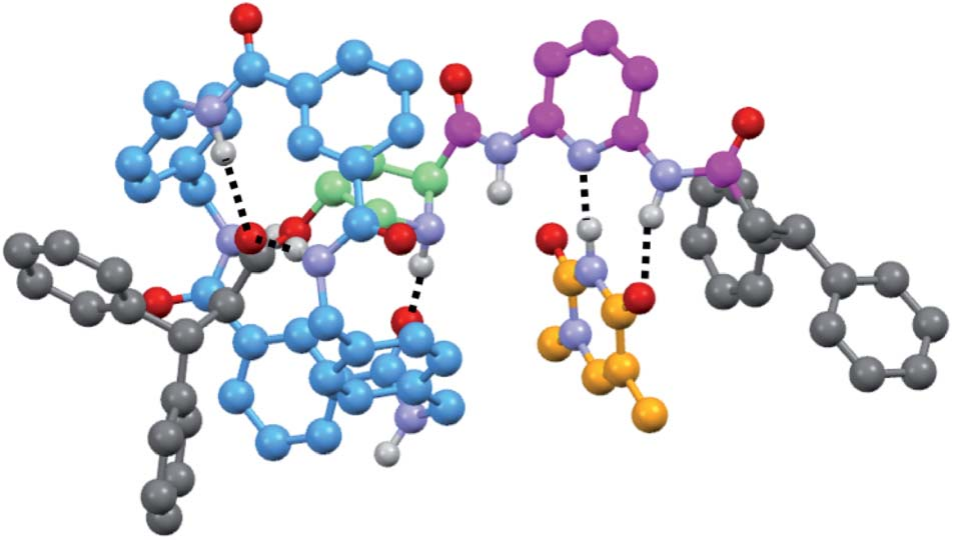
Computed minimum-energy co-conformer of the supramolecular complex **6a·T′** (with T′ = *N*^1^-methylthymine) at the M06/cc-pVDZ theoretical level.

The DAP functionality is able to interact with neutral molecules, such as barbiturates, flavins or thymine derivatives, *via* a recognition process by forming a complementary DAD–ADA hydrogen bonding network.^20^ At this point, we reasoned that the DAP unit in **6** could enable the formation of a supramolecular complex with a suitable guest inducing the translation of the ring to the proline ester frame (see Fig. 2). In this regard, we next explored the ability of rotaxanes **6** to interact with *N*-hexylthymine (**T**) by calculating the association constant of the formed 1 : 1 complex.^14*a*^ We found that this cofactor is able to compete with the ring for the DAP unit in the thread. Titration ^1^H NMR experiments (CD_2_Cl_2_, 298 K) showed that **6a,b** are able to bind to **T** with similar association constants of ∼20 M^−1^ through the DAP unit (see the ESI, Fig. S2–S5†). To further confirm this weak association we carried out ^1^H PGSE (Pulsed Gradient Spin Echo) diffusion measurements on solutions of **T** and **6b** in CDCl_3_ at 298 K, revealing an 8% decrease of the D coefficient of the thymine derivative as a consequence of its complexation with the rotaxane (see the ESI, Tables S7 and S8†).

Note that the association constants of **6·T** are lower than those of other reported similar complexes,^14^ the proline fragment being the only differential structural fragment. For further information, we computed the **6a·T′** (with T′ = *N*^1^-methylthymine) supramolecular aggregate at the DFT level (Fig. 2) in which the ring simultaneously interacts with the ester and the amino group of the pyrrolidine core (see the ESI†). The formation of only two out of three possible H-bonds between the DAP and thymine units, probably to favour aromatic interactions with the nearby diphenylmethyl group, accounts for the moderate strength of this interaction. It should be noted that the estimated magnitude of the association constants of **6·T** are in the same order as that obtained for the free thread **4** (22 M^−1^) (see the ESI, Fig S6 and S7†), reinforcing the idea of the disturbing effect of the proximal five-membered ring on the H-bonding DAD array.

### 2.2 Background reactivity of the thread **4** and rotaxane **6a** in the presence of acetone

**Scheme 2:**
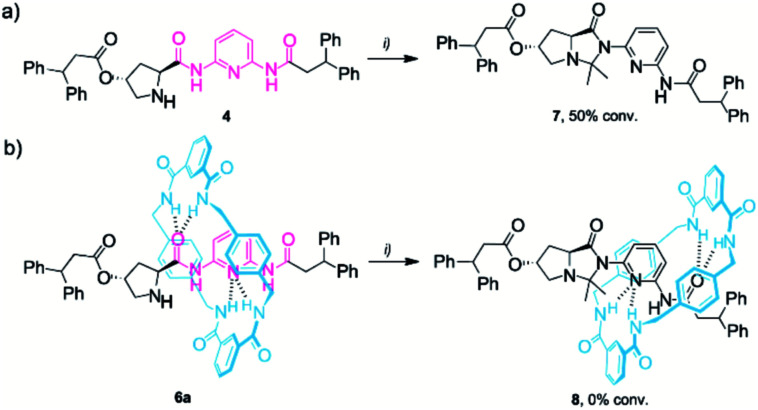
Formation of the imidazolidone derivative of (a) thread **4** and (b) rotaxane **6a**. Reaction conditions: (i) acetone (20 equiv.), CDCl_3_ (0.025 M), 25 °C, 2 days. The full experimental procedures can be found in the ESI.†

A characteristic scenario found in enamine-type transformations catalysed by pyrrolidine-based systems is catalyst deactivation due to undesirable side reactions. The formation of cyclic species obtained by the intramolecular attack of the corresponding enamine on various side groups of the catalyst, such as secondary amides or carboxyl groups, is often reported.^21^ The presence of such relatively stable species generally results in a decrease of the reaction rate (low conversion) and selectivity (low e. r.). This is why we monitored the stability of thread **4** and rotaxane **6a** in the presence of an excess of acetone (Scheme 2). We found that after 48 h, around 50% of the thread **4** was consumed, affording the cyclic imidazolidone **7** (Scheme 2a and Fig. S8†). Importantly, compound **7**, easily isolable and characterised by HRMS (ESI^+^), showed a moderate stability in solution. After 72 h in CDCl_3_ at room temperature, 70% conversion of **7** into the initial thread **4** was observed, releasing free acetone (ESI, Fig. S9†). In stark contrast, rotaxane **6a** remained completely unaffected when subjected to the same reaction conditions as the mechanical bond precludes the formation of the undesired cyclic byproduct **8** (Scheme 2b).

### 2.3 Catalytic activity of the interlocked prolinamides

**Fig. 3:**
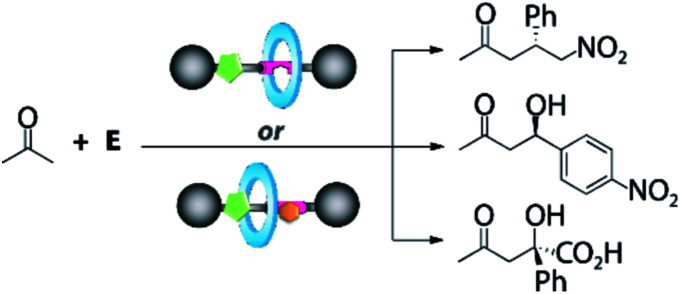
Selected enamine-type processes studied in this work.

Once the DAP-based rotaxanes **6** were assembled, we decided to explore their aptitude as organocatalysts by comparing their activity with that of the free thread **4**. For this study we chose three enamine-type transformations: aldol reactions between acetone and *p*-nitrobenzaldehyde^22^ or phenylglyoxylic acid^23^ and the Michael addition of acetone to β-nitrostyrene (Fig. 3).^24^ Testing the activity of *N*-hexylthymine as a cofactor was also planned.

#### 2.3.1 Asymmetric Michael addition of acetone to β-nitrostyrene

After a short optimization of the reaction conditions (see the ESI, Tables S2 and S3†), we decided to use dichloromethane as the solvent, as it should allow the establishment of intercomponent hydrogen bonds between the thread and the macrocycle, thus precluding random ring motion in the interlocked structures. As we expected, thread **4** was shown to be inactive (Table 1, entry 1). This lack of reactivity was attributed to the formation of the inactive cyclic imidazolidone **7**, which can be easily formed due to the large molar excess of acetone (ratio of acetone : **4**, 100 : 1). In contrast moderate conversions were achieved when rotaxanes **6a,b** were used, although affording adduct **9** in a poor e. r. (Table 1, entries 2 and 3).

**Table 1:** Organocatalyzed Michael addition of acetone to β-nitrostyrene^a^

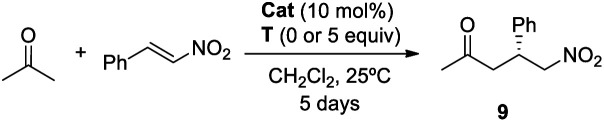				
Entry	Cat	**T**	% conv^b^	e. r.^c^
1	**4**	NO	<5	—
2	**6a**	NO	55	57 : 43
3	**6b**	NO	28	54 : 46
4	**4**	YES	17	57 : 43
5	**6a**	YES	85	78 : 22
6	**6b**	YES	95	91 : 9
7	—	YES	0	0

a Reaction conditions: acetone (0.25 mmol), β-nitrostyrene (0.025 mmol), catalyst (10 mol%), *N*-hexylthymine (0 or 0.125 mmol), CH_2_Cl_2_ (100 μL), 25 °C, 5 days.

b Determined by ^1^H NMR from the crude reaction.

c e. r. determined by HPLC using a Daicel ChiralPak AS-H column.

Interestingly, the presence of up to 5 equivalents of *N*-hexylthymine (**T**) in the reaction media positively altered the course of the process (Table 1, entries 4–6; see the ESI for further details, Table S5†) whereas other complementary cofactors (barbital and non-alkylated thymine) did not cause similar pronounced changes (see the ESI, Table S4†). It should be noted that the reaction carried out in the presence of **T** without the catalyst was fully unproductive (Table 1, entry 7). In the case of thread **4**, a slight increase of the conversion towards adduct **9** was detected (Table 1, compare entries 1 and 4), although it was practically unselective (57 : 43 e. r.). It is probable that the presence of **T** in the reaction media precludes the formation of **7** increasing the productivity of **4** for the formation of **9**. In contrast, the more constrained rotaxanes **6** showed both better activity and selectivity. It is important to highlight that the enhancement of the catalytic behaviour of the interlocked systems compared with the free thread is a rather uncommon effect, as the macrocycle usually inhibits or reduces the activity of the functionalities settled inside or near its cavity.^25^ Note that the major reason for this overall outcome could lie in the inhibition of **4** by the substrate (see Scheme 2). Remarkably, rotaxane **6b**, with nitro groups at the macrocycle, was observed to be the best catalyst, achieving nearly full conversion and remarkably increasing the enantiomeric ratio to 91 : 9 e. r. of the adduct **9** instead of the poor e. r. in the absence of **T** (Table 1, compare entries 3 and 6). Note that the acidity of the amide NH protons of the macrocycle in **6b** is slightly increased by the electron-withdrawing NO_2_ groups. In this scenario it seems reasonable that an intermolecular hydrogen bonding interaction macrocycle-electrophile could be established, where the ring acts as a second activation site, similar to a bifunctional catalyst.^26^ This performance is noteworthy: catalyst **6b**, which initially is a poorly active and completely unselective system, is converted to an enhanced supramolecular catalyst **6b·T** upon addition of a complementary cofactor. Additional control experiments (see the ESI, Table S6†) carried out using the non-interlocked macrocycle as the catalyst, alone or in combination with **4** and/or **T**, demonstrated the need for the mechanical bond to obtain good activities and selectivities during the considered Michael addition.

#### 2.3.2 Asymmetric aldol reaction of acetone with *p*-nitrobenzaldehyde

In this reaction thread **4** again displayed extremely poor activity, showing low conversion towards adduct **10** and moderate enantioselectivity (Table 2, entry 1), which is in agreement with the undesired but favourable formation of the imidazolidone **7**. Moreover, a high amount of enone^27^**11**, formed in the competitive condensation pathway, was also observed (ratio of **10** : **11**, 50 : 50). Remarkably, higher conversions were obtained when the rotaxanes **6a,b** were used and, more importantly, the formation of the enone **11** was almost avoided (Table 2, entries 2 and 3).

**Table 2:** Organocatalyzed aldol reaction of acetone with *p*-nitrobenzaldehyde^a^

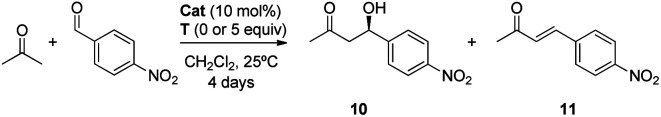					
Entry	Cat	**T**	% conv^b^	**10** : **11**^c^	e. r.^d^
1	**4**	NO	14	50 : 50	76 : 24
2	**6a**	NO	58	91 : 9	76 : 24
3	**6b**	NO	60	100 : 0	88 : 12
4	**4**	YES	76	83 17	62 : 38
6	**6b**	YES	78	91 : 9	70 : 30

a Reaction conditions: acetone (0.25 mmol), *p*-nitrobenzaldehyde (0.025 mmol), catalyst (10 mol%), *N*-hexylthymine (0 or 0.125 mmol), CH_2_Cl_2_ (100 μL), 25 °C, 4 days.

b Determined by ^1^H NMR from the crude reaction.

c Ratio of **10** : **11** was determined by ^1^H NMR analysis.

d e. r. determined by HPLC using a Daicel ChiralPak AS-H column.

As in prior results, the nitro-containing rotaxane **6b** was the best catalyst, improving the conversion to adduct **10** (60%), the chemoselectivity (enone **11** was not detected) and the enantioselectivity (88 : 12 e. r.) of the process (Table 2, entry 3). The presence of the mechanical bond apparently creates a well-defined chiral environment where the enamine intermediate is located, inside which the new C–C bond forming reaction occurs, generating enantioenriched products with higher selectivities.

In this transformation the addition of thymine as the cofactor was unfruitful, lowering the enantiomeric ratios, although displaying a slight increase of the conversion (Table 2, entries 4–6). Apparently, in this reaction the activating interaction of macrocycle-electrophile is better established in the initial rotaxane than in the thymine-cofactoring state.

#### 2.3.3 Asymmetric aldol reaction of acetone with phenylglyoxylic acid

Finally, thread **4** and rotaxanes **6** were shown to be highly active in catalysing this second aldol reaction (Table 3, entries 1–3). The great activity shown by thread **4** in this case could probably be explained by the establishment of hydrogen-bond interactions between the acid group of the electrophile and the basic nitrogen atom of the pyridine.^23*a*,28^ As in previous examples, the selectivity shown by thread **4** was lower than those shown by the constrained rotaxanes, with rotaxane **6b** again giving the best results (almost full conversion and a high 92 : 8 e. r. for aldol **12** after 48 hours). When thymine **T** is present the e. r. decreased (Table 3, entry 5).

**Table 3:** Organocatalyzed aldol reaction of acetone with phenylglyoxylic acid^a^

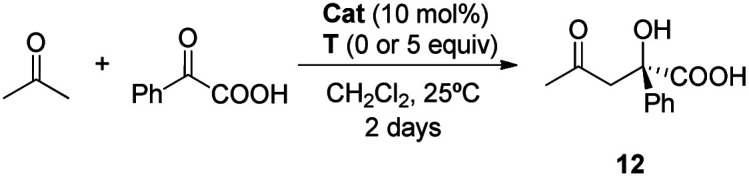				
Entry	Cat	**T**	% conv^b^	e. r.^c^
1	**4**	NO	93	75 : 25
2	**6a**	NO	54	89 : 11
3	**6b**	NO	85	92 : 8
4	**4**	YES	100	60 : 40
5	**6b**	YES	38	72 : 28

a Reaction conditions: acetone (0.25 mmol), phenylglyoxylic acid (0.025 mmol), catalyst (10 mol%), *N*-hexylthymine (0 *or* 0.125 mmol), CH_2_Cl_2_ (100 μL), 25 °C, 2 days.

b Determined by ^1^H NMR from the crude reaction.

c e. r. determined by HPLC using a Daicel ChiralPak AS-H column. For analytical reasons, the adduct **12** was derivatized to its methyl ester (see the ESI).

### 2.4 Exploring the activity of the catalyst **6b** in competitive experiments

**Scheme 3:**
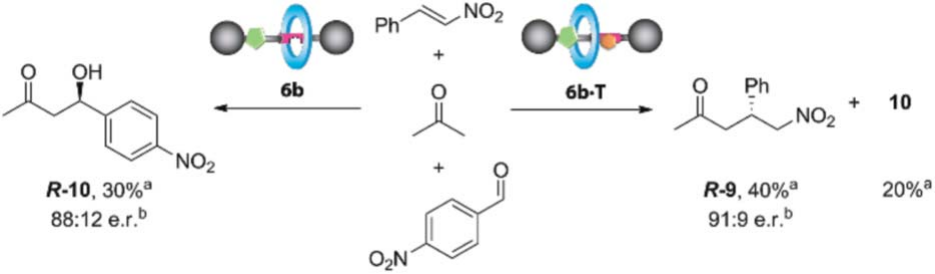
Michael *versus* aldol addition of acetone using rotaxane **6b** as the catalyst in the presence or absence of *N*-hexylthymine (**T**). Reaction conditions: *p*-nitrobenzaldehyde (1 equiv.), *trans*-β-nitrostyrene (1 equiv.), acetone (1.5 equiv.), catalyst **6b** (10 mol%), *N*-hexylthymine (5 equiv., if required), CH_2_Cl_2_ (0.25 M), 25 °C, 5 days.^a^ Determined by ^1^H NMR from the crude reaction. ^b^ e. r. determined by HPLC using a Daicel ChiralPak AS-H column.

Catalyst **6b** clearly works under two different regimes in the studied processes, either uncomplexed or complexed with *N*-hexylthymine forming the supramolecular interlocked catalyst **6b·T**. Thus, we envisioned that the addition or not of the cofactor **T** could influence the distribution rate between the final adducts when two electrophiles are simultaneously present. Therefore, we carried out competitive experiments by adding *p*-nitrobenzaldehyde (1 equiv.) and β-nitrostyrene (1 equiv.) to acetone (1.5 equiv.) in the presence of catalyst **6b** (10 mol%), with or without **T** (Scheme 3).

After 5 days, we observed the formation of the aldol adduct **10** (30%), with only traces of the Michael adduct **9** (<5%) when **T** was not added. In contrast, the chemoselectivity became reversed in the presence of **T**, preferentially forming the Michael adduct **9** (40%), (ratio of **9** : **10**, 2 : 1) (see the ESI, Fig. S10†). Importantly, the isolated adducts **9** and **10** maintained the enantiomeric ratios previously obtained in the individual experiments.

## 3. Conclusions

In summary, we have synthesized a series of chiral mechanically interlocked diacylaminopyridine-based prolinamides in a straightforward manner (only 4 synthetic steps). Their catalytic activity was modulated by complexation with a complementary DAD array (*N*-hexylthymine), forming a supramolecular catalyst. Importantly, the presence of the flexible and, at the same time, bulky isophthalamide ring improves the ability of the interlocked systems as catalysts when compared with the free thread, by creating a dynamic chiral pocket. As a result, the mechanical bond precludes the formation of inactive species that inhibit the catalysis, thus enhancing the overall catalytic activity of the interlocked systems. This remarkable behaviour differs from the considerably lower catalytic activity shown by previously reported rotaxane-based catalysts when compared with their non-interlocked threads. In our interlocked catalysts the benzylic amide macrocycle has not only a shielding effect but also an activating role. The presence of electron-withdrawing groups attached to the macrocycle, which increase the acidity of the NH amide groups, is beneficial for the outcomes of the assayed reactions, usually increasing the conversion and enantioselectivities. The addition or not of an external cofactor to the reaction media allows switching of the chemoselectivity in competitive experiments. All these facts allow this versatile system to work in two dissimilar regimes, being an effective catalyst in three different enamine-type processes.

These results highlight that the employment of mechanically interlocked molecules as catalysts could open exciting paths in the field of asymmetric catalysis thus making a contribution to the design of new systems that could tackle challenging transformations.

## Conflicts of interest

There are no conflicts to declare.

## Supplementary Material

SC-011-D0SC00444H-s001
