# Supplementary material for: Enhancing the selectivity of prolinamide organocatalysts using the mechanical bond in [2]rotaxanes
Source: Chem Sci. 2020 Mar 11;11(14):3629–35. doi: 10.1039/d0sc00444h (PMC8152698; doi:10.1039/d0sc00444h)
Supplement: SC-011-D0SC00444H-s001 [file SC-011-D0SC00444H-s001.pdf]

Electronic Supplementary Material (ESI) for Chemical Science.
This journal is © The Royal Society of Chemistry 2020

Enhancing the Selectivity of Prolinamide Organocatalysts by the Mechanical Bond in [2]Rotaxanes

María Calles,[a] Julio Puigcerver,[a] Diego A. Alonso,[b] Mateo Alajarin,[a] Alberto Martinez-Cuezva[a,]* and Jose Berna[a,]*

[a] Departamento de Química Orgánica, Facultad de Química, Regional Campus of International Excellence "Campus Mare Nostrum", Universidad de Murcia, E-30100 Murcia, Spain

[b] Departamento Química Orgánica, Facultad de Ciencias, Universidad de Alicante, E-03080, Alicante, Spain

E-mail: amcuezva@um.es
ppberna@um.es

Table of Contents…………………………………………………………………………………… S2

1. General experimental section………………………………………………………………… S3

2. Synthesis of thread 3 ..…………………………………………………………………………… S4

3. General procedure for the preparation of [2]rotaxanes 5 ………………………………………... S5

4. Boc-deprotection of thread 3 …………………………………………………………………….. S7

5. Boc-deprotection of rotaxanes 5 ……...………………………………………………………… S8

6. Stacked ^{1}H NMR spectra of thread 4 and [2]rotaxane 6a ……………………………………….. S9

7. Analysis of the proton chemical shift of He in the DAP derivatives 4 and 6a…………………… S10

8. Titration experiments of rotaxane 6a,b and thread 4 with *N*-hexylthymine …………………… S10

9. Michael reaction between acetone and β-nitrostyrene: Optimization of the reaction conditions S13

10. General procedures under the optimized conditions ………….…………………………….. S16

11. Evaluation of the stability of thread 4 and rotaxane 6a in the presence of acetone …………… S17

12. Competitive experiments with catalyst 6b …………………………………………………….. S19

13. Computational Studies for the complex 6a:T ………………………………………………… S20

14. DOSY NMR experiments for *N*-hexylthymine and rotaxane 6b ……………………………….. S22

15. ^{1}H and ^{13}C NMR spectra of synthesized compounds ……………….……………………………… S24

16. Selected Copies of HPLC Traces of Synthesized Compounds ……………….…………………….. S33

1. General Experimental Section

Unless stated otherwise, all reagents were purchased from Aldrich Chemicals and used without further purification. HPLC grade solvents (Scharlab) were nitrogen saturated and were dried and deoxygenated using an Innovative Technology Inc. Pure-Solv 400 Solvent Purification System. Column chromatography was carried out using silica gel (60 Å, 70-200 µm, SDS) as stationary phase, and TLC was performed on precoated silica gel on aluminun cards (0.25 mm thick, with fluorescent indicator 254 nm, Fluka) and observed under UV light. All melting points were determined on a Kofler hot-plate melting point apparatus and are uncorrected. ^{1}H- and ^{13}C-NMR spectra were recorded on a Bruker Avance 300, 400 and 600 MHz instruments. ^{1}H NMR chemical shifts are reported relative to Me_4Si and were referenced via residual proton resonances of the corresponding deuterated solvent whereas ^{13}C NMR spectra are reported relative to Me_4Si using the carbon signals of the deuterated solvent. Signals in the ^{1}H and ^{13}C NMR spectra of the synthesized compounds were assigned with the aid of DEPT, APT, or two-dimensional NMR experiments (COSY, HMQC and HMBC). Abbreviations of coupling patterns are as follows: br, broad; s, singlet; d, doublet; t, triplet; q, quadruplet; m, multiplet. The deuterated solvent $CDCl_3$ was filtered through a pad of Na_2CO_3 and dried with molecular sieves prior to use. Coupling constants *(J)* are expressed in Hz. High-resolution mass spectra (HRMS) were obtained using a time-of-flight (TOF) instrument equipped with electrospray ionization (ESI). Optical rotation ($[\alpha]_D^{25}$) was measured with a JASCO P-1020 polarimeter (concentration: g/mL in chloroform as solvent). The enantiomeric ratios were determined by HPLC analysis employing a chiral stationary phase column specified in the individual experiment, by comparing the samples with the appropriate racemic mixtures. Elemental analysis were measured in a LECO TruSpec Micro CHNS Analyzer.

Abbreviation list:

DMAP: dimethylaminopyridine

EDCI: *N*-(3-dimethylaminopropyl)-*N'*-ethylcarbodiimide hydrochloride

DIPEA: *N,N*-diisopropylethylamine

HOBt: hydroxybenzotriazole

DMF: *N,N*-dimethylformamide

TFA: trifluoroacetic acid

DAP: diacylaminopyridine

TLC: thin layer chromatography

2. Synthesis of thread 3

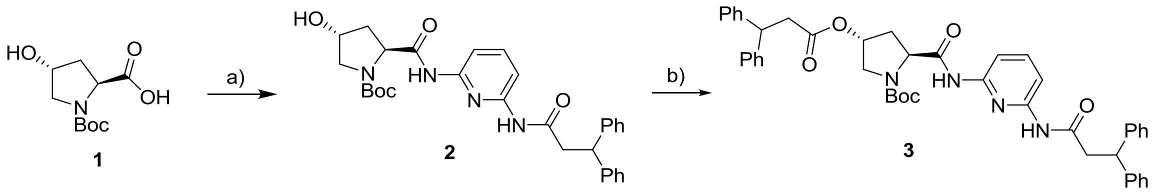

Scheme S1. a) Fragment **S1**, EDCI, DIPEA, HOBt, CH_2Cl_2, 0°C to r.t., overnight; c) **S1**, EDCI, DMAP, CH_2Cl_2, 0°C to r.t., overnight.

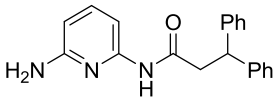

S1

Fragment **S1** was synthesized following the described procedure reported in A. Martinez-Cuezva, J. Berna, R.-A. Orenes, A. Pastor, M. Alajarin, *Angew. Chem. Int. Ed.*, **2014**, *53*, 6762-6767 and showed identical spectroscopic data as those reported therein.

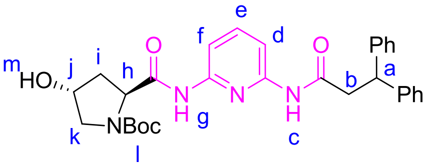

2

To a solution of *N*-Boc-protected *trans*-hydroxy- L-proline **1** (362.7 mg, 1.57 mmol) and Et_3N (0.22 mL, 1.57 mmol) in dry THF (50 mL) under N_2 atmosphere at 0ºC was added dropwise ethyl chloroformate (0.15 mL, 1.57 mmol) during 15 min. After stirring for 30 min at 25ºC, fragment **S1** (0.5 g, 1.57 mmol) was added. The mixture was stirred overnight at room temperature. After this time the reaction was refluxed for 3h. The solvent was removed under reduced pressure and AcOEt (20 mL) was added. The suspension was filtered and the filtrate was washed with brine (2 × 20 mL). The organic phase was dried over anhydrous $MgSO_4$ and concentrated under reduced pressure. The solid crude was subjected to column chromatography on silica gel using hexane/AcOEt (from 1/2 to 1/3) mixture as eluent to give the title product as a white solid (**2**, 490 mg, 59%); mp 112-114 ºC; $[\alpha]_D^{25}$ – 6.4° (*c* 0.0106, $CHCl_3$); ^{1}H NMR (400 MHz, $CDCl_3$, 318K) δ 9.06 (s, 1H, H_g), 8.05 (s, 1H, H_c), 7.80-7.74 (m, 2H, H_{f+d}), 7.57 (t, 1H, $J = 7.9$ Hz, H_e), 7.28-7.24 (m, 8H, Ph), 7.20-7.14 (m, 2H, Ph), 4.68 (t, 1H, $J = 7.7$ Hz, H_a), 4.61-4.53 (m, 1H, H_h), 4.53-4.45 (m, 1H, H_j), 3.76-3.50 (m, 2H, H_k), 3.13 (d, 2H, $J = 7.7$ Hz, H_b), 2.83 (s, 1H, H_m), 2.48-3.05 (m, 2H, H_i), 1.43 (s, 9H, H_l) ppm; ^{13}C NMR (100 MHz, $CDCl_3$, 318 K) δ 170.9 (CO), 170.0 (CO), 155.8 (CO) 149.6 (C), 149.3 (C), 143.7 (C), 140.9 (CH), 128.8 (CH), 127.9 (CH), 126.8 (CH), 109.9 (CH), 109.6 (CH), 81.4 (C), 70.0 (CH), 60.4 (CH), 55.3 (CH_2), 47.2 (CH), 44.2 (CH_2), 28.5 (CH_3); HRMS (ESI) calcd for $C_{30}H_{35}N_4O_5$ $[M + H]^+$ 531.2602, found 531.2610.

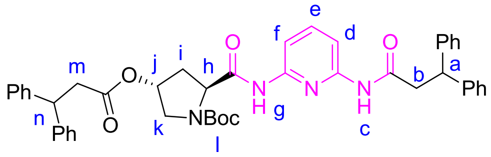

3

To a solution of compound **2** (2.49 g, 4.69 mmol) and Et_3N (0.67 mL, 4.69 mmol) in dry dichloromethane (50 mL) under N_2 atmosphere at 0ºC was added 3,3-diphenylpropanoyl chloride (1.22 g, 5 mmol) dropwise. The reaction mixture was stirred at room temperature overnight. After this time the reaction mixture was washed with a saturated solution of $NaHCO_3$ (2 × 50 mL) and brine (2 × 50 mL). The organic phase was dried over anhydrous $MgSO_4$ and concentrated under reduced pressure. The solid crude was subjected to column chromatography on silica gel using hexane/AcOEt (from 1/2 to 1/4) mixture as eluent to give the title product as a white solid (**3**, 2.18 g, 63%); mp 68-70 ºC; $[\alpha]_D^{25}$ – 10.2° (*c* 0.0097, $CHCl_3$); ^{1}H NMR (400 MHz, $CDCl_3$, 318 K) δ 8.65 (s, 1H, H_g), 7.83-7.74 (m, 2H, H_{d+f}), 7.2 (t, *J* = 8.2 Hz, 1H, H_e), 7.44 (s, 1H, H_c), 7.34-7.16 (m, 20H, Ph), 5.21-5.17 (m, 1H, H_j), 4.68 (t, *J* = 7.6 Hz, 1H, H_a), 4.51 (t, *J* = 8.1 Hz, 1H, H_n), 4.13 (s, 1H, H_h), 3.60-3.45 (m, 2H, H_k), 3.14-3.06 (m, 4H, H_{b+m}), 2.48-2.18 (m, 1H, H_i), 2.08-1.88 (m, 1H, H_i), 1.48 (s, 9H, H_l) ppm; ^{13}C NMR (100 MHz, $CDCl_3$, 318 K) δ 171.2 (CO), 169.9 (CO), 169.5 (CO), 149.6 (CO), 149.4 (C), 143.7 (C), 143.3 (C), 143.3 (C), 140.6 (CH), 128.8 (CH), 127.9 (CH), 127.8 (CH), 126.9 (CH), 126.9 (CH), 126.8 (CH), 109.9 (CH), 109.6 (CH), 81.4 (C), 72.7 (CH), 59.9 (CH_2), 52.4 (CH_2), 47.5 (CH), 47.3 (CH), 44.3 (CH_2), 41.0 (CH_2), 28.5 (CH_3); HRMS (ESI) calcd for $C_{45}H_{47}N_4O_6$ $[M + H]^+$ 739.3490, found 739.3478.

3. General procedure for the preparation of [2]rotaxanes 5

The thread (1 equiv.) and Et_3N (24 equiv.) in anhydrous $CHCl_3$ (300 mL) were stirred vigorously whilst solutions of *p*-xylylenediamine (8 equiv.) in anhydrous $CHCl_3$ (20 mL) and the corresponding isophthaloyl chloride (8 equiv.) in anhydrous $CHCl_3$ (20 mL) were simultaneously added over a period of 4 h using motor-driven syringe pumps. After a further 4 h the resulting suspension was filtered through a Celite® pad, washed with water (2 × 50 mL), an aqueous solution of HCl 1N (2 × 50 mL), a saturated solution of $NaHCO_3$ (2 × 50 mL) and brine (2 × 50 mL). The organic phase was dried over $MgSO_4$ and the solvent removed under reduced pressure. The resulting solid was subjected to column chromatography (silica gel) to yield unconsumed thread and the [2]rotaxane.

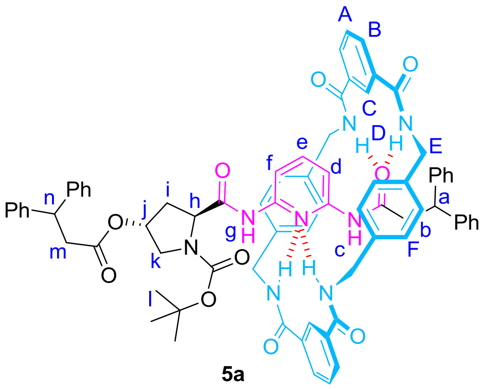

5a

Rotaxane **5a** was obtained following the described method from thread **3** (1.00 g, 1.35 mmol), *p*-xylylendiamine (1.5 g, 10.8 mmol) and isophthaloyl chloride (2.0 g, 10.8 mmol). The solid crude was subjected to column chromatography on silica gel using a $CHCl_3$/MeOH (30:1) mixture as eluent to give the title product as a white solid (**5a**, 257 mg, 15%); mp 123-125 ºC; $[\alpha]_D^{25}$ – 38.4° (*c* 0.01, $CHCl_3$); ^{1}H NMR (400 MHz, $CDCl_3$, 318 K) δ 8.36 (s, 2H, H_C), 8.28-8.20 (m, 4H, H_B), 7.80 (s, 2H, NH_D), 7.60 (t, *J* = 7.7 Hz, 2H, H_A), 7.43-7.10 (m, 23H, Ph + $H_{d+c+g+e+f}$), 7.06-6.95 (m, 2H, Ph), 6.85 (d, *J* = 7.8 Hz, 4H, H_F), 6.68 (d, *J* = 7.8 Hz, 4H, H_F), 6.52 (s, 2H, NH_D), 5.12-5.04 (m, 1H, H_j), 4.75-4.64 (m, 2H, H_E), 4.50 (t, *J* = 8.0 Hz, 1H, H_n), 4.44 (s, 4H, H_E), 4.16 (bs, 1H, H_a), 4.08-3.97 (m, 2H, H_E), 3.71-3.58 (m, 1H, H_h), 3.46 (dd, *J* = 12.3, 5.5 Hz, 1H, H_k), 3.30 (dd, *J* = 12.3, 3.7 Hz, 1H, H_k), 3.09 (d, *J* = 8.0 Hz, 2H, H_m), 2.73-2.48 (m, 2H, H_b), 2.40-2.26 (m, 1H, H_i), 1.81-1.71 (m, 1H, H_i), 1.46 (s, 9H, H_l) ppm; ^{13}C NMR (100 MHz, $CDCl_3$, 318 K) δ 171.2 (CO), 167.7 (CO), 166.4 (CO), 148.9 (CO), 143.9 (C), 143.5 (C), 143.3 (C), 137.2 (C), 137.0 (C), 134.5 (C), 132.0 (CH), 131.5 (CH), 129.3 (CH), 129.1 (CH), 128.9 (CH), 128.9 (CH), 128.6 (CH), 127.8 (CH), 127.7 (CH), 127.0 (CH), 127.0 (CH), 126.9 (CH), 126.8 (CH), 125.7 (CH), 109.6 (CH), 82.5 (C), 72.1 (CH), 59.0 (CH), 52.3 (CH_2), 47.4 (CH), 46.7 (CH), 45.0 (CH_2), 44.4 (CH_2), 42.6 (CH_2), 41.0 (CH_2), 28.7 (CH_3) ppm; HRMS (ESI) calcd for $C_{77}H_{75}N_8O_{10}$ $[M + H]^+$ 1271.5601, found 1271.5626.

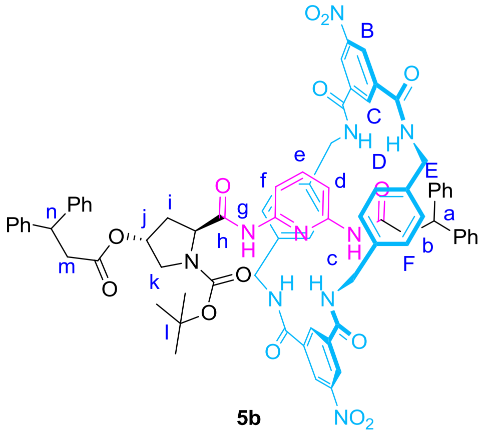

5b

Rotaxane **5b** was obtained following the described method from thread **3** (1.2 g, 1.62 mmol), *p*-xylylendiamine (1.8 g, 13.0 mmol) and 4-nitroisophthaloyl chloride (2.8 g, 13.0 mmol). The solid crude was subjected to column chromatography on silica gel using a $CHCl_3$/MeOH (30:1) mixture as eluent to

give the title product as a white solid (**5b**, 397 mg, 18%); mp 138-140 ºC; $[\alpha]_D^{25}$ – 63.6° (*c* 0.011, $CHCl_3$); ^{1}H NMR (400 MHz, $CDCl_3$, 318 K) δ 9.02-8.97 (m, 4H, H_B), 8.50 (s, 2H, H_C), 8.17 (s, 2H, NH_D), 8.03 (s, 1H, NH_g), 7.44-7.41 (M, 2H, H_{d+f}), 7.35-7.12 (m, 19H, Ph + He), 7.10-7.06 (m, 2H, Ph), 7.00-6.95 (m, 5H, H_F+ NH_c), 6.72 (d, *J* = 7.7 Hz, 4H, H_F), 6.13 (s, 2H, NH_D), 5.12-5.07 (m, 1H, H_j), 4.67-4.52 (m, 5H, H_{E+n}), 4.45 (dd, *J* = 14.7, 4.4 Hz, 2H, H_E), 4.20 (t, *J* = 7.4 Hz, 1H, H_a), 4.06 (dd, *J* = 14.3, 3.3 Hz, 2H, H_E), 3.47 (t, *J* = 6.8 Hz, 1H, H_h), 3.39 (d, *J* = 3.8 Hz, 2H, H_k), 3.20 (d, *J* = 8.1, 1.4 Hz, 2H, H_m), 2.86 (dd, *J* = 16.6, 8.0 Hz, 1H, H_b), 2.72 (dd, *J* = 16.6, 6.8 Hz, 1H, H_b), 2.48-2.40 (m, 1H, H_i), 1.83-1.75 (m, 1H, H_i), 1.50 (s, 9H, H_l) ppm; ^{13}C NMR (100 MHz, $CDCl_3$, 318 K) δ 173.0 (CO), 172.1 (CO), 168.8 (CO), 166.6 (CO), 164.7 (CO), 157.5 (C), 149.7 (CO), 149.4 (C), 149.2 (C), 144.5 (C), 144.3 (C), 144.1 (C), 144.0 (C), 141.3 (CH), 137.9 (C), 137.1 (C), 137.0 (C), 136.9 (C), 131.7 (CH), 129.8 (CH), 129.7 (CH), 129.6 (CH), 129.4 (CH), 129.2 (CH), 128.6 (CH), 128.5 (CH), 128.3 (CH), 128.2 (CH), 127.8 (CH), 127.7 (CH), 127.5 (CH), 127.4 (CH), 127.0 (CH), 126.5 (CH), 110.6 (CH), 109.6 (CH), 82.9 (C), 72.7 (CH), 60.0 (CH), 53.5 (CH_2), 48.0 (CH), 47.5 (CH), 45.8 (CH_2), 45.3 (CH_2), 43.0 (CH_2), 41.3 (CH_2), 34.0 (CH_2), 29.5 (CH_3) ppm; HRMS (ESI) calcd for $C_{77}H_{73}N_{10}O_{14}$ $[M + H]^+$ 1361.5302, found 1361.5312.

4. Boc-deprotection of thread 3

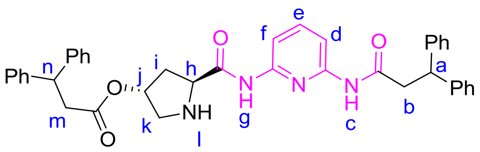

4

To a solution of Boc-protected thread **3** (300 mg, 0.405 mmol) in chloroform (20 mL) was added TFA (0.31 mL, 4.05 mmol). The reaction was stirred at room temperature overnight. After this time, the reaction mixture was washed with a saturated solution of aqueous $NaHCO_3$ (2 × 20 mL) and brine (2 × 20 mL). The organic phase was dried over anhydrous $MgSO_4$ and concentrated under reduced pressure, to give the title product as a white solid (**4**, 245 mg, 95 %); mp 90-92 ºC; $[\alpha]_D^{25}$ + 7.2° (*c* 0.0022, $CHCl_3$); ^{1}H NMR (300 MHz, $CDCl_3$, 298 K) δ 9.72 (s, 1H, H_g), 7.87-7-76 (m, 2H, H_{d+f}), 7.67 (s, 1H, H_c), 7.63 (t, *J* = 8.1 Hz, 1H, H_e), 7.36-7.08 (m, 20H, Ph), 5.11-5.02 (m, 1H, H_j), 4.70 (t, *J* = 7.7 Hz, 1H, H_a), 4.50 (t, *J* = 8.4 Hz, 1H, H_n), 3.75 (t, *J* = 8.4 Hz, 1H, H_h), 3.10-3.02 (m, 4H, H_{b+m}), 2.77-2.61 (m, 2H, H_k), 2.26-2.14 (m, 1H, H_i), 2.02-1.80 (m, 2H, H_i + NH_l) ppm; ^{13}C NMR (75 MHz, $CDCl_3$, 298 K) δ 172.9 (CO), 171.4 (CO), 169.5 (CO), 149.5 (C), 149.1 (C), 143.4 (C), 143.2 (C), 140.8 (CH), 128.8 (CH), 128.7 (CH), 127.9 (CH), 127.8 (CH), 127.6 (CH), 127.0 (CH), 126.8 (CH), 109.7 (CH), 109.4 (CH), 60.5 (CH), 53.0 (CH_2), 47.8 (CH), 47.0 (CH), 44.2 (CH_2), 41.1 (CH_2), 36.3 (CH_2) ppm; HRMS (ESI) calcd for $C_{40}H_{39}N_4O_4$ [M +

H]$^+$ 639.2966, found 639.2956; Elemental analysis: Calc. (Found) C 75.21 (75.49), H 6.00 (6.196), N 8.77 (8.566).

5. Boc-deprotection of rotaxanes 5

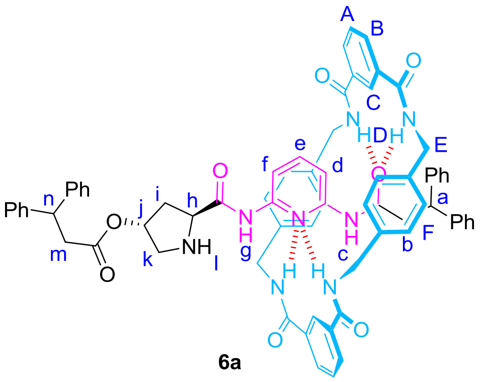

6a

To a solution of Boc-protected rotaxane **5a** (166 mg, 0.13 mmol) in chloroform (20 mL) was added TFA (0.10 mL, 1.30 mmol). The reaction was stirred at room temperature overnight. After this time the reaction mixture was diluted with chloroform (20 mL) and washed with a saturated solution of aqueous $NaHCO_3$ (2 × 20 mL) and brine (2 × 20 mL). The organic phase was dried over anhydrous $MgSO_4$ and concentrated under reduced pressure, to give the title product as a white solid (**6a**, 144 mg, 95 %); mp 162-164 ºC; $[\alpha]_D^{25}$ + 42° (*c* 0.0098, $CHCl_3$); ^{1}H NMR (300 MHz, $CDCl_3$, 298 K) δ 9.33 (s, 1H, H_g), 8.92 (s, 1H, H_c), 8.56 (s, 2H, H_C), 8.22 (d, J = 7.8 Hz, 2H, H_B), 8.13 (d, J = 7.8 Hz, 2H, H_B), 7.57 (t, J = 7.8 Hz, 2H, H_A), 7.53-7.30 (m, 7H, H_{d+e+f} + NH_D), 7.25-7.04 (m, 20H, Ph), 6.75-6.66 (m, 8H, H_F), 4.62 (dd, J = 14.3, 5.8 Hz, 2H, H_E), 4.52 (t, J = 7.8 Hz, 1H, H_a), 4.44-4.22 (m, 5H, H_{E+n}), 4.15-3.96 (m, 3H, H_{E+j}), 3.10 (d, J = 7.8 Hz, 2H, H_b), 2.98-2.83 (m, 3H, H_{h+m}), 1.85-1.75 (m, 1H, H_k), 1.55-1.35 (m, 2H, H_{k+i}), 1.15-1.05 (m, 1H, H_i) ppm; ^{13}C NMR (75 MHz, $CDCl_3$, 298 K) δ 174.9 (CO), 172.5 (CO), 171.9 (CO), 167.7 (CO), 167.2 (CO), 151.0 (C), 149.2 (C), 144.3 (C), 143.9 (C), 143.8 (C), 141.5 (CH), 138.2 (C), 138.1 (C), 135.5 (C), 135.5 (C), 132.5 (CH), 132.0 (CH), 130.4 (CH), 129.7 (CH), 129.6 (CH), 128.6 (CH), 128.5 (CH), 127.9 (CH), 127.8 (CH), 127.6 (CH), 125.3 (CH), 112.0 (CH), 110.3 (CH), 127.5 (CH), 127.4 (CH), 126.4 (CH), 110.5 (CH), 110.3 (CH), 74.8 (CH), 59.4 (CH), 51.3 (CH_2), 48.1 (CH), 48.0 (CH), 44.9 (CH_2), 44.7 (CH_2), 44.4 (CH_2), 41.5 (CH_2), 35.7 (CH_2) ppm; HRMS (ESI) calcd for $C_{72}H_{67}N_8O_8$ [M + H]$^+$ 1171.5076, found 1171.5057; Elemental analysis: Calc. (Found) C 73.83 (73.88), H 5.68 (5.828), N 9.57 (9.612).

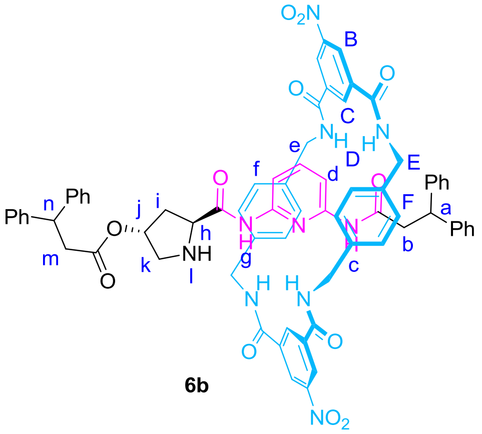

6b

To a solution of Boc-protected rotaxane **5b** (368.1 mg, 0.271 mmol) in chloroform (20 mL) was added TFA (0.42 mL, 5.40 mmol). The reaction was stirred at room temperature overnight. After this time, the reaction mixture was diluted with chloroform (20 mL) and washed with a saturated solution of aqueous $NaHCO_3$ (2 × 20 mL) and brine (2 × 20 mL). The organic phase was dried over anhydrous $MgSO_4$ and concentrated under reduced pressure, to give the title product as a white solid (**6b**, 327 mg, 96 %); mp 158-160 ºC; $[\alpha]_D^{25}$ + 64° (*c* 0.0217, $CHCl_3$); ^1H NMR (400 MHz, CD_2Cl_2, 298 K) δ 9.04 (s, 1H, NH_g), 9.01 (s, 2H, H_C), 8.95 (s, 2H, H_B), 8.91 (s, 2H, H_B), 8.66 (s 1H, NH_c), 7.70-7.56 (m, 5H, NH_D +H_e), 7.46 (d, 1H, H_d), 7.32-7.10 (m, 21H, H_f + Ph), 6.86-6.75 (m, 8H, H_F), 4.71 (dd, J = 14.4, 5.8 Hz, 2H, H_E), 4.55-4.20 (m, 7H, $H_{E+a+n+j}$), 4.12-4.03 (m, 2H, H_E), 3.17 (d, J = 7.8 Hz, 2H, H_m), 3.00-2.80 (m, 3H, H_{b+h}), 1.80-1.55 (m, 2H, H_k), 1.40-1.05 (m, 2H, H_l) ppm; ^{13}C NMR (100 MHz, CD_2Cl_2, 298 K) δ 175.0 (CO), 171.8 (CO), 171.1 (CO), 164.5 (CO), 164.4 (CO), 150.1 (C), 149.6 (C), 148.9 (C), 143.8 (C), 143.8 (C), 143.7 (C), 141.7 (CH), 137.8 (C), 137.6 (C), 136.7 (C), 136.5 (C), 130.0 (C), 129.4 (CH), 129.3 (CH), 129.2 (CH), 129.2 (CH), 129.1 (CH), 129.1 (CH), 128.0 (CH), 127.9 (CH), 127.9 (CH), 127.3 (CH), 126.6 (CH), 126.2 (CH), 111.5 (CH), 110.4 (CH), 75.0 (CH), 59.4 (CH), 51.3 (CH_2), 47.6 (CH), 47.5 (CH), 44.6 (CH_2), 44.4 (CH_2), 43.7 (CH_2), 40.8 (CH_2), 35.7 (CH_2); HRMS (ESI) calcd for $C_{72}H_{65}N_{10}O_{12}$ $[M + H]^+$ 1261.4778, found 1261.4759; Elemental analysis: Calc. (Found) C 68.56 (68.33), H 5.11 (5.116), N 11.10 (11.18).

6. Stacked ^1H NMR spectra of thread 4 and [2]rotaxane 6a

We investigated the ring location over the thread once the interlocked structure **6a** is assembled by ^1H NMR experiments. The comparison of the ^1H NMR spectra of the thread **4** and rotaxane **6a** recorded in $CDCl_3$ showed that the signals of to the pyridine ring (H_d, H_e and H_f) are shifted to higher field in the rotaxane in -0.21 ppm. The shielding of the pyrrolidine core is also appreciable by the upfield shifting of the signals of its protons: $\Delta\delta(H_j)$ = -1.12 ppm; $\Delta\delta(H_h)$ = -0.88 ppm; $\Delta\delta(H_k)$ = -1.68 ppm; $\Delta\delta(H_i)$ = -0.88 ppm.

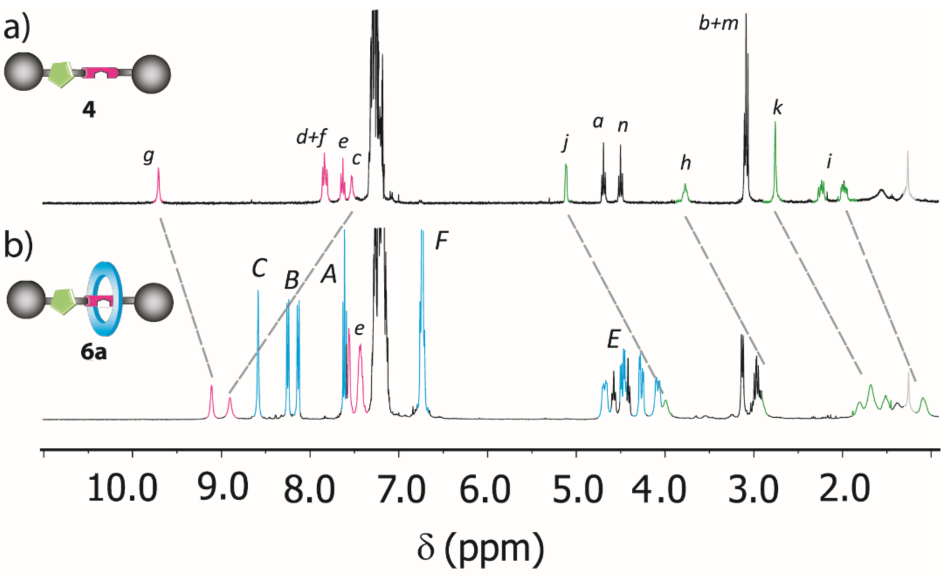

Figure S1. ^{1}H NMR spectra (400 MHz, $CDCl_3$, 298 K) of: a) thread **4**; b) [2]rotaxane **6a**. lettering change

7. Analysis of the proton chemical shift of H_e in the DAP derivatives 4 and 6a

In order to analyze the location of the macrocycle over the thread in the rotaxane **6a** we focused our attention on the variation of the chemical shift of the hydrogen atom at 4-position of the pyridine ring (H_e) when the rotaxane is assembled. This value was compared with the variation of the shift of this proton in rotaxane **B** and its precursor thread **A** (Table S1).

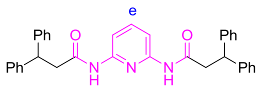

A

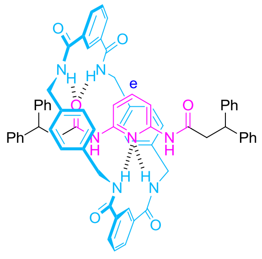

B

Table S1. Chemical shifts of the H_e signal in DAP-based systems.

Entry	Compound	δ(H_e) ppm	Δδ(H_e) ppm
1	**A**	7.61[a]	0.25
2	**B**	7.36[a]	
3	**4**	7.63	0.21
4	**6a**	7.42	
[a] Chemical shifts reported in: A. Martinez-Cuezva *et al*, *Angew. Chem., Int. Ed.* **2014**, *53*, 6762–6767.			

8. Titration experiments of rotaxane 6a,b and thread 4 with *N*-hexylthymine

^{1}H NMR titration spectra were recorded on a Bruker Avance 400 MHz spectrometer, in CD_2Cl_2 at 298 K.

Method for the titration with *N*-hexylthymine (**T**): A solution of *N*-hexylthymine (40 mM, and 2 mM in **host**) was added to a solution of host (rotaxanes **6a, 6b** or thread **4**) (0.5 mL, 2 mM). The chemical shift

of a specific host proton NH_c was monitored for seventeen titration points (for 0.0-20.0 equivalents of added guest).

Rotaxane 6a with *N*-hexylthymine (T)

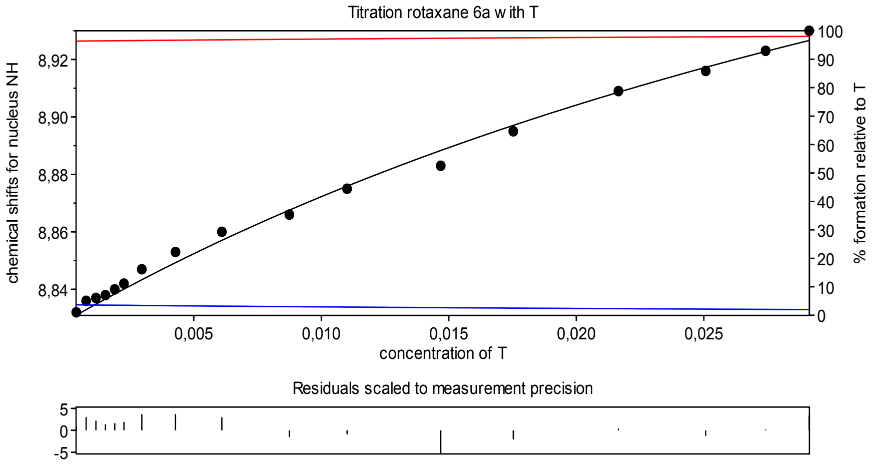

Figure S2. HypNMR 2008 fitting data for *N*-hexylthymine binding to rotaxane **6a** followed by ^{1}H NMR spectra (400 MHz, CD_2Cl_2, 298 K)

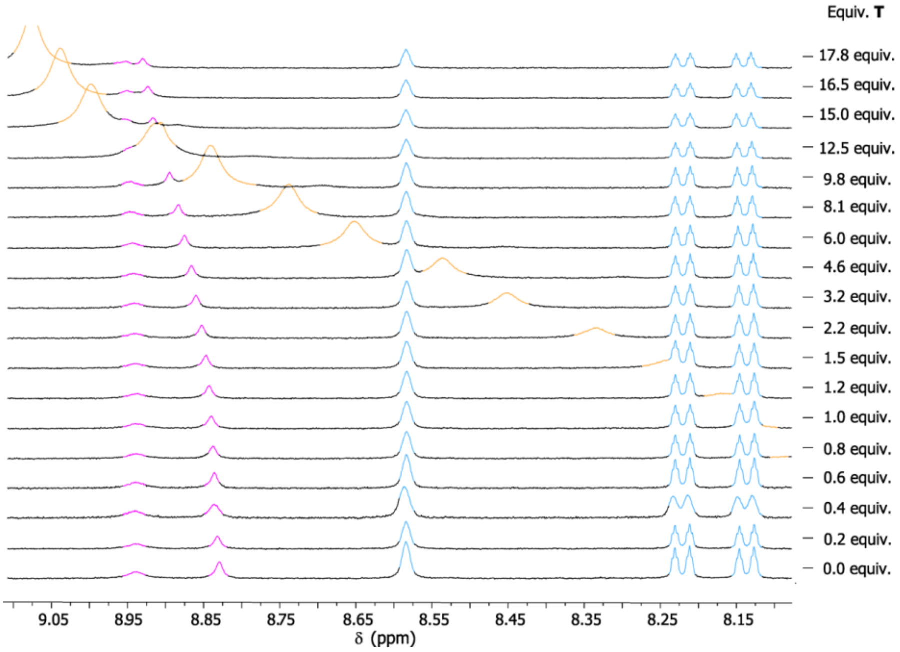

Figure S3. Partial ^{1}H NMR spectra of titration of rotaxane **6a** with *N*-hexylthymine (400 MHz, CD_2Cl_2, 298 K). Chemical shift of amide proton NH_{c+g} (pink) in the presence of *N*-hexylthymine (orange).

Rotaxane 6b with *N*-hexylthymine (T)

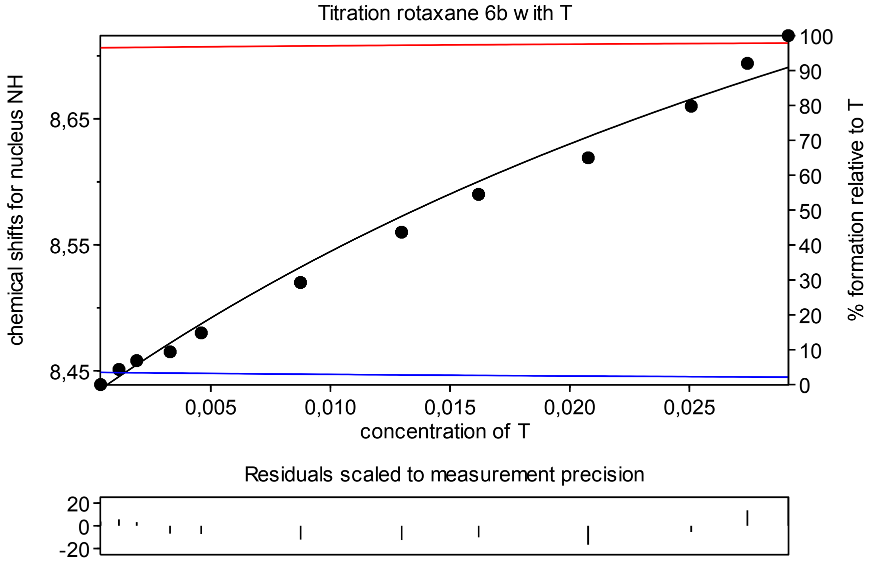

Figure S4. HypNMR 2008 fitting data for *N*-hexylthymine binding to rotaxane **6b** followed by ^{1}H NMR spectra (400 MHz, CD_2Cl_2, 298 K)

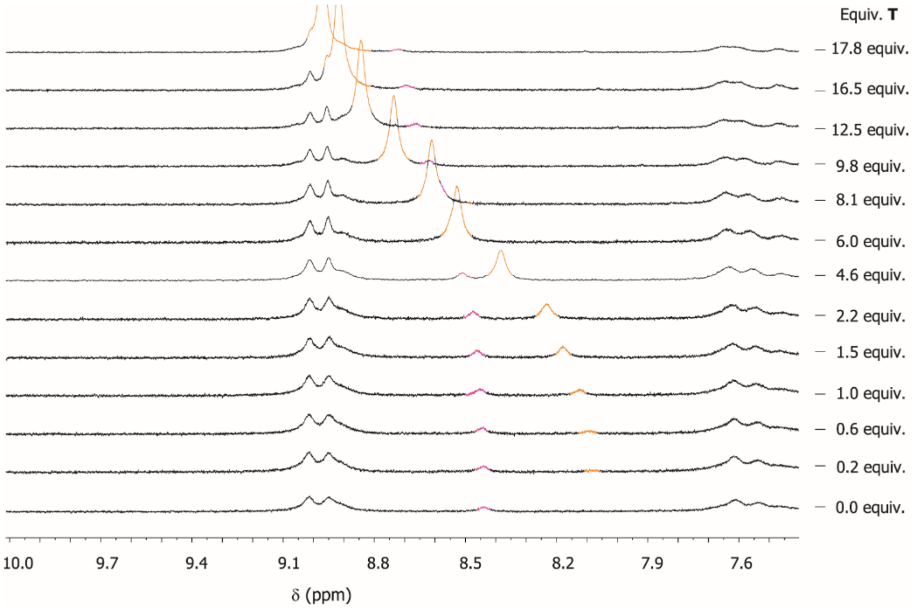

Figure S5. Partial ^{1}H NMR spectra of titration of rotaxane **6b** with *N*-hexylthymine (400 MHz, CD_2Cl_2, 298 K). Chemical shift of amide proton NH_{c+g} (pink) in the presence of *N*-hexylthymine (orange).

Thread 4 with *N*-hexylthymine (T)

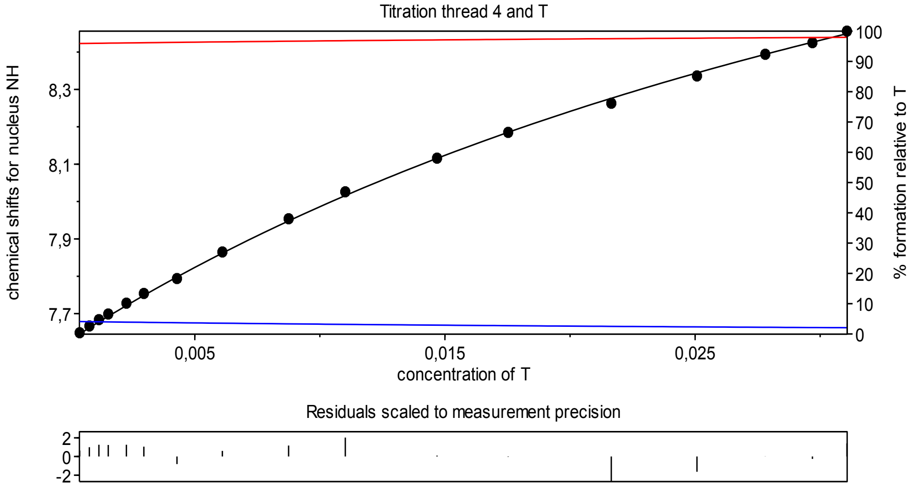

Figure S6. HypNMR 2008 fitting data for *N*-hexylthymine binding to thread **4** followed by ^{1}H NMR spectra (400 MHz, CD_2Cl_2, 298 K)

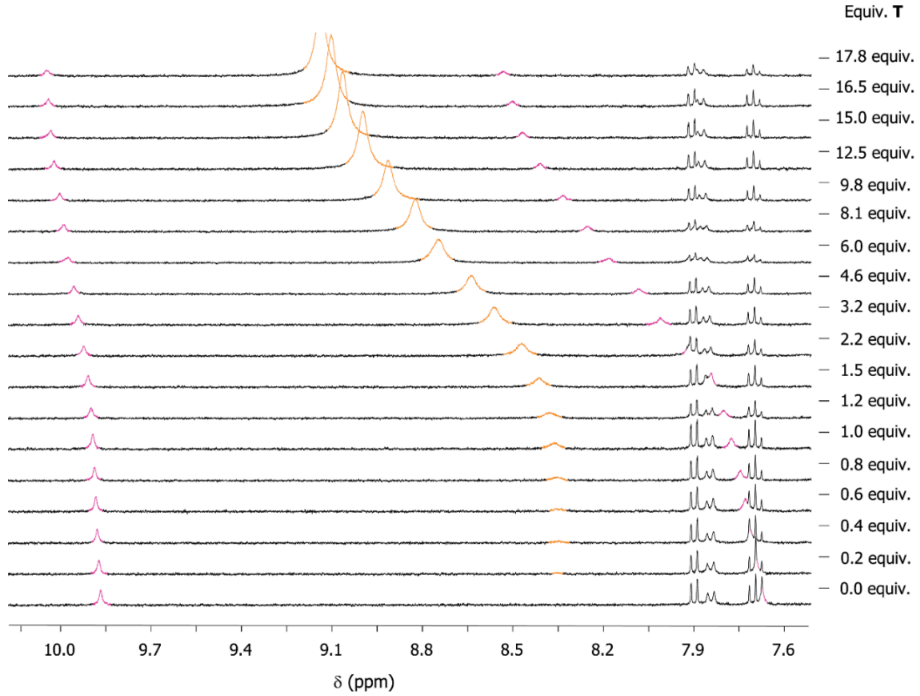

Figure S7. Partial ^{1}H NMR spectra of titration of thread **4** with *N*-hexylthymine (400 MHz, CD_2Cl_2, 298 K). Chemical shift of amide proton NH_{c+g} (pink) in the presence of *N*-hexylthymine (orange).

9. Michael reaction between acetone and β-nitrostyrene. Optimization of the reaction conditions

The asymmetric Michael reaction between acetone and β-nitrostyrene in the presence of catalytic amounts of the suitable catalyst (10 mol%) was tested. Different additives and conditions were screened for the optimization of the process.

Table S2. Solvent screening.[a]

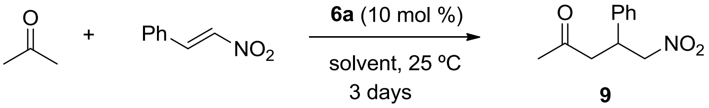

entry	Solvent	Conv. (%)[b]	e.r.[c]	Configuration
1	CH_2Cl_2	55	**57**:43	*S*
2	acetone	21	50:50	-
3	DMF	17	46:**54**	*R*
4	THF	-	-:-	-
5	MeCN	< 5	-:-	-

[a]Reaction conditions: β-nitrostyrene (0.025 mmol), acetone (18 µL), **6a** (10 mol%), solvent (100 µL), 3 days; [b]Calculated by ^{1}H NMR; [c]Determined by HPLC with a ASH chiral stationary phase.

Table S3. Catalyst screening.[a]

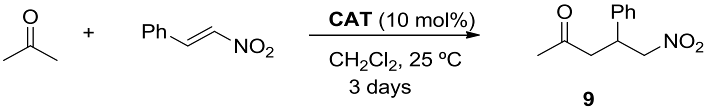

entry	CAT	Conv. (%)[b]	e.r.[c]	Configuration
1	**4**	<5	-:-	-
2	**6a**	55	57:43	*S*
3	**6b**	28	54:46	*S*

[a]Reaction conditions: β-nitrostyrene (0.025 mmol), acetone (18 µL), **catalyst** (10 mol%), CH_2Cl_2 (100 µL), 25 °C, 3 days; [b]Calculated by ^{1}H NMR; [c]Determined by HPLC with a ASH chiral stationary phase.

Table S4. Additive screening: *DAD arrays*.[a]

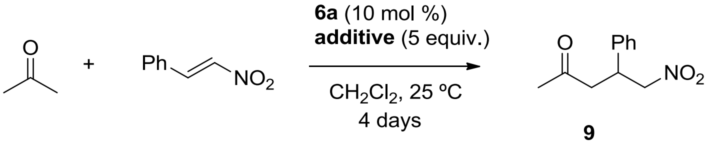

entry	Additive	Conv. (%)[b]	e. r.[c]	Configuration
1	*N*-hexylthymine (**T**)	47	33:67	*R*
2	thymine	29	55:45	*S*
3	barbital	40	48:52	*R*
4[d]	*N*-hexylthymine (**T**)	-	-	-

[a]Reaction conditions: β-nitrostyrene (0.025 mmol), acetone (18 µL, 10 equiv.), **6a** (10 mol%), CH_2Cl_2 (100 µL), additive (1 equiv.), 25 °C, 4 days; [b]Calculated by ^{1}H NMR; [c]Determined by HPLC with a ASH chiral stationary phase; [d] Without catalyst **6a**.

Table S5. Additive screening: *N*-hexylthymine.[a]

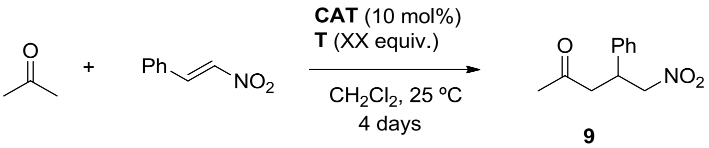

entry	CAT	T (equiv.)	Conv. (%)[b]	e. r.[c]	Configuration
1	**4**	0.1	20	49:51	*R*
2	**4**	5	17	43:57	*R*
3	**6a**	0.1	25	45:55	*R*
4	**6a**	1	47	33:67	*R*
5	**6a**	2	80	27:73	*R*
6	**6a**	5	85	22:78	*R*
7	**6b**	2	72	13:87	*R*
8	**6b**	5	95	9:**91**	*R*
9	**6b**	10	77	14:86	*R*

[a]Reaction conditions: β-nitrostyrene (0.025 mmol), acetone (18 μL, 10 equiv.), catalyst (10 mol%), *N*-hexylthymine (**xx** equiv.), CH_2Cl_2 (100 μL), 25 °C, 4 days; [b]Calculated by ^1H NMR; [c]Determined by HPLC with a ASH chiral stationary phase.

Table S6. Evaluation of the non-interlocked components as catalysts.[a]

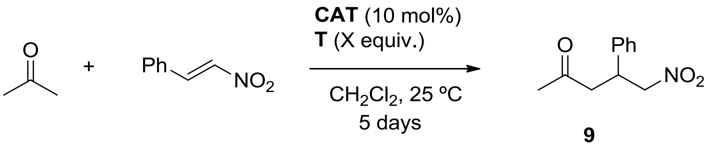

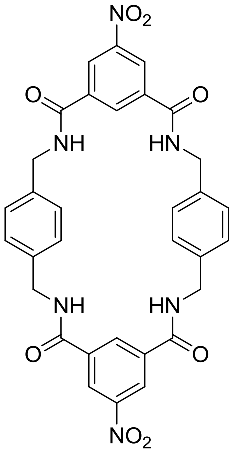

MAC

entry	CAT	T (equiv.)	Conv. (%)[b]	e. r.[c]	Configuration
1	**MAC**[d]	-	-	-:-	-
2	**4+MAC**	-	<5	-:-	-
3	**4+MAC**	5	30	44:56	*R*

[a]Reaction conditions: β-nitrostyrene (0.025 mmol), acetone (18 μL, 10 equiv.), catalyst (10 mol%), *N*-hexylthymine (**xx** equiv.), CH_2Cl_2 (100 μL), 25 °C, 4 days; [b]Calculated by ^1H NMR; [c]Determined by HPLC with a ASH chiral stationary phase;[d] Syntheized following the method described in: A. Martinez-Cuezva, L. V. Rodrigues, C. Navarro, F. Carro-Guillen, L. Buriol, C. P. Frizzo, M. A. P. Martins, M. Alajarin and J. Berna, *J. Org. Chem.*, 2015, **80**, 10049–10059.

10. General procedures under the optimized conditions

Michael addition between acetone and *trans*-nitrostyrene: A solution of the β-nitrostyrene (0.025 mmol), acetone (0.25 mmol), *N*-hexylthymine (0.125 mmol) and rotaxane **6b** (10 mol%) in dry CH_2Cl_2 (100 μL) at room temperature was stirred for a period of 5 days. After this time, pentane was added and the suspension was filtered through a pad of Celite® to remove the catalyst. The filtrate was concentrated under vacuum and analyzed by ^1H NMR spectroscopy for the calculation of the conversion. The desired Michael adduct **9** was purified by preparative TLC and the enantiomeric excess analyzed by chiral HPLC.
NOTE: The racemate was synthesized employing pyrrolidine (30 mol%) as catalyst.

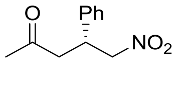

(*R*)-9

Compound **9** was described in H. Huang, E. N. Jacobsen, *J. Am. Chem. Soc.* **2006**, *128*, 7170; and showed identical spectroscopic data as those reported therein. The enantiomeric ratio was determined by HPLC analysis using Daicel Chiralpak AS-H; hexane:iPrOH = 75:25, 1 mL/min, λ = 254 nm: t_r = 10.5 min (*S* enantiomer), t_r = 12.6 min (*R* enantiomer). The HPLC protocol was described in the same reference, where the two enantiomers are identified.

Aldol addition between acetone and *p*-nitrobenzaldehyde: A solution of the *p*-nitrobenzaldehyde (0.025 mmol), acetone (0.25 mmol) and rotaxane **6b** (20 mol%) in dry CH_2Cl_2 (100 μL) at room temperature was stirred for a period of 5 days. After this time, pentane was added and the suspension was filtered through a pad of Celite® to remove the catalyst. The filtrate was concentrated under vacuum and analyzed by ^1H NMR spectroscopy for the calculation of the conversion and selectivity. The desired aldol adduct **10** was purified by preparative TLC and the enantiomeric excess analyzed by chiral HPLC.
NOTE: The racemate was synthesized employing pyrrolidine (30 mol%) as catalyst.

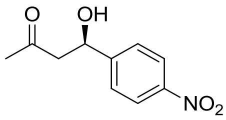

(*R*)-10

Compound **10** was described in Z. Tang, F. Jiang, L.-T. Yu, X. Cui, L.-Z. Gong, A.-Q. Mi, Y.-Z. Jiang, Y.-D. Wu, *J. Am. Chem. Soc.* **2003**, *125*, 5262; and showed identical spectroscopic data as those reported therein. The enantiomeric ratio was determined by HPLC analysis using Daicel Chiralpak AS-H; hexane:iPrOH = 70:30, 1 mL/min, λ = 254 nm: t_r = 8.2 min (*R* enantiomer), t_r = 10.3 min (*S* enantiomer). The HPLC protocol was described in the same reference, where the two enantiomers are identified.

Aldol addition between acetone and phenylglyoxylic acid: A solution of the phenylglyoxylic acid (0.025 mmol), acetone (0.25 mmol) and rotaxane **6b** (10 mol%) in dry CH_2Cl_2 (100 μL) at room temperature was stirred for a period of 2 days. After this time, pentane was added and the suspension was filtered through a pad of Celite® to remove the catalyst. The filtrate was concentrated under vacuum and analyzed by ^{1}H NMR spectroscopy for the calculation of the conversion to adduct **12**. To the reaction crude a solution of $TMSCHN_2$ in hexane (25 μL) was added and the reaction stirred for 30 min. The reaction was concentrated under vacuum and the desired aldol adduct **12Me** was purified by preparative TLC and the enantiomeric excess analyzed by chiral HPLC.
NOTE: The racemate was synthesized employing pyrrolidine (30 mol%) as catalyst.

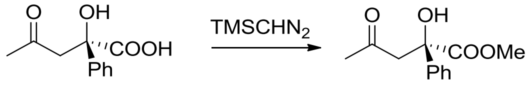

(*R*)-12 **(*R*)-12Me**

Compound **12Me** was described in Z. Tang, L.-F. Cun, X. Cui, A.-Q. Mi, Y.-Z. Jiang, L.-Z. Gong, *Org. Lett.* **2006**, *8*, 1263; and showed identical spectroscopic data as those reported therein. The enantiomeric ratio was determined by HPLC analysis using Daicel Chiralpak AS-H; hexane:iPrOH = 70:30, 1 mL/min, λ = 254 nm: t_r = 5.6 min (*S* enantiomer), t_r = 6.3 min (*R* enantiomer). The HPLC protocol was described in the same reference, where the two enantiomers are identified.

11. Evaluation of the stability of thread 4 and rotaxane 6a in the presence of acetone

We evaluated the stability of catalysts **4** and **6a** in the presence of acetone. These systems are prompted to react with acetone, forming a imidazolidone derivative, which is less active in enamine-type transformations. Thus, we followed up the changes of these systems by ^{1}H NMR spectroscopy.

General procedure: Thread **4** or rotaxane **6a** were dissolved in dry $CDCl_3$ (0.4 mL, 25 mM) and acetone was added (20 equiv.). The formation of the corresponding imidazolidone derivative (**7** or **8**) was followed over time. The rotaxane **6a** showed high stability, not observing the formation of the imidazolidone **8** in any case.

In order to isolate the imidazolidone **7** we carried out a further experiment: Thread **4** (30 mg) was dissolved in acetone (1 mL) and $CHCl_3$ (1 mL). Activated 3Å molecular sieves (100 mg) were added and the solution was stirred at 40 ºC during 24 hours. After this time, solvent was removed under reduced pressure to give the imidazolidone **7** as a yellow solid (29 mg).

NOTE: a minor amount of thread **4** is observed at the beginning, which is increasing over time due to the instability of the imidazolidone **7**.

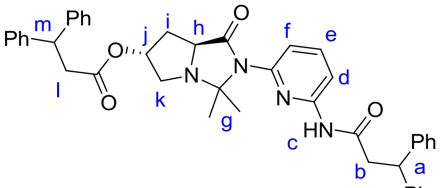

7

^{1}H NMR (400 MHz, $CDCl_3$, 298 K) δ 7.89 (d, J = 8.1 Hz, 1H, H_d), 7.65 (t, J = 8.1 Hz, 1H, H_e), 7.63 (s, 1H, NH_c), 7.37 (dd, J = 0.7, 8.0 Hz, 1H, H_d), 7.32-7.18 (m, 20H, Ph), 5.17-5.12 (m, 1H, H_j), 4.64 (t, J = 7.7 Hz, 1H, H_a), 4.57 (t, J = 8.2 Hz, 1H, H_m), 3.99 (dd, J = 3.8, 9.9 Hz, 1H, H_h), 3.17 (d, J = 7.7 Hz, 1H, H_b), 3.11 (d, J = 8.2 Hz, 1H, H_m), 2.92 (dd, J = 1.9, 10.9 Hz, 1H, H_i), 2.64 (dd, J = 4.1, 10.9 Hz, 1H, H_i), 2.49 (ddd, J = 3.8, 6.8, 15.3 Hz, 1H, H_k), 2.03-1.95 (m, 1H, H_k), 1.75 (s, 3H, H_g), 1.44 (s, 3H, H_g) ppm; HRMS (ESI) calcd for $C_{43}H_{43}N_4O_4$ $[M + H]^+$ 679.3279, found 679.3259.

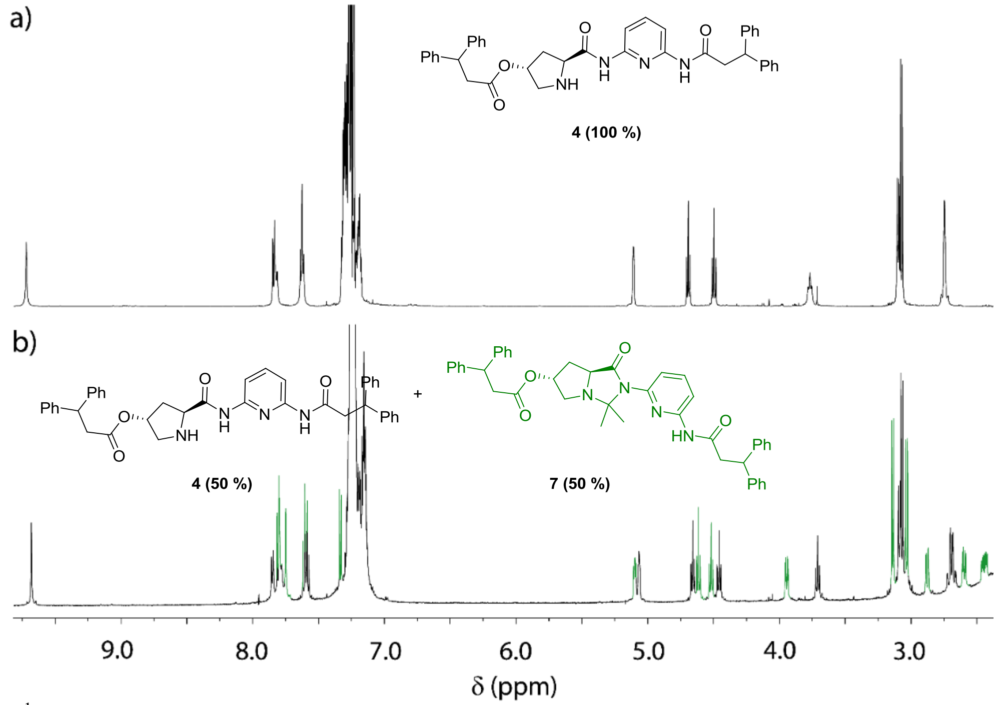

Figure S8. Partial ^{1}H NMR (600 MHz, $CDCl_3$, 298 K) of thread **4** in the presence of 20 equivalents of acetone: a) at time 0 h; b) after 48 h.

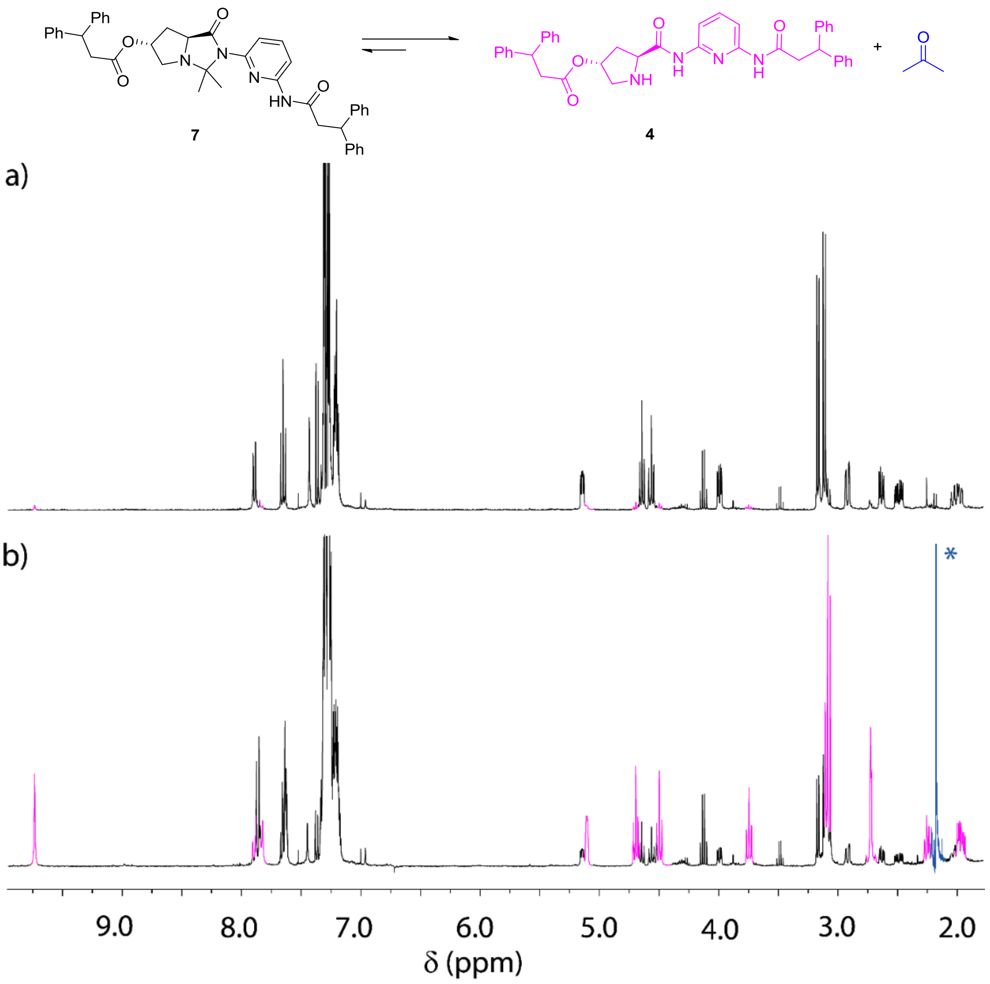

Figure S9. Partial ^{1}H NMR (400 MHz, $CDCl_3$, 298 K) of isolated imidazolidone **7**: a) time 0 h; b) after 72 h (acetone: *).

12. Competitive experiments with catalyst 6b

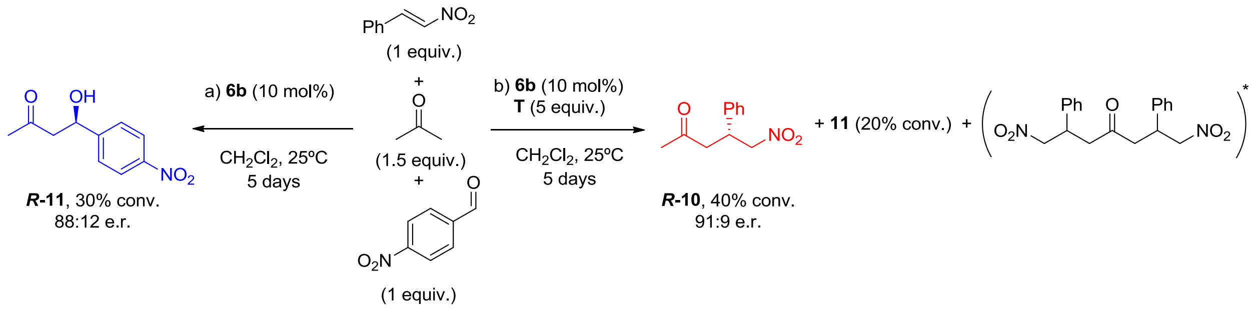

Scheme S2. Michael *versus* aldol addition of acetone using rotaxane **6b** as catalyst in the presence or not of *N*-hexylthymine (**T**). Reaction conditions: *p*-nitrobenzaldehyde (1 equiv.), *trans*-β-nitrostyrene (1 equiv.), acetone (1.5 equiv.), catalyst **6b** (10 mol%), *N*-hexylthymine (5 equiv., *if required*), CH_2Cl_2 (0.25 M), 25 °C, 5 days.

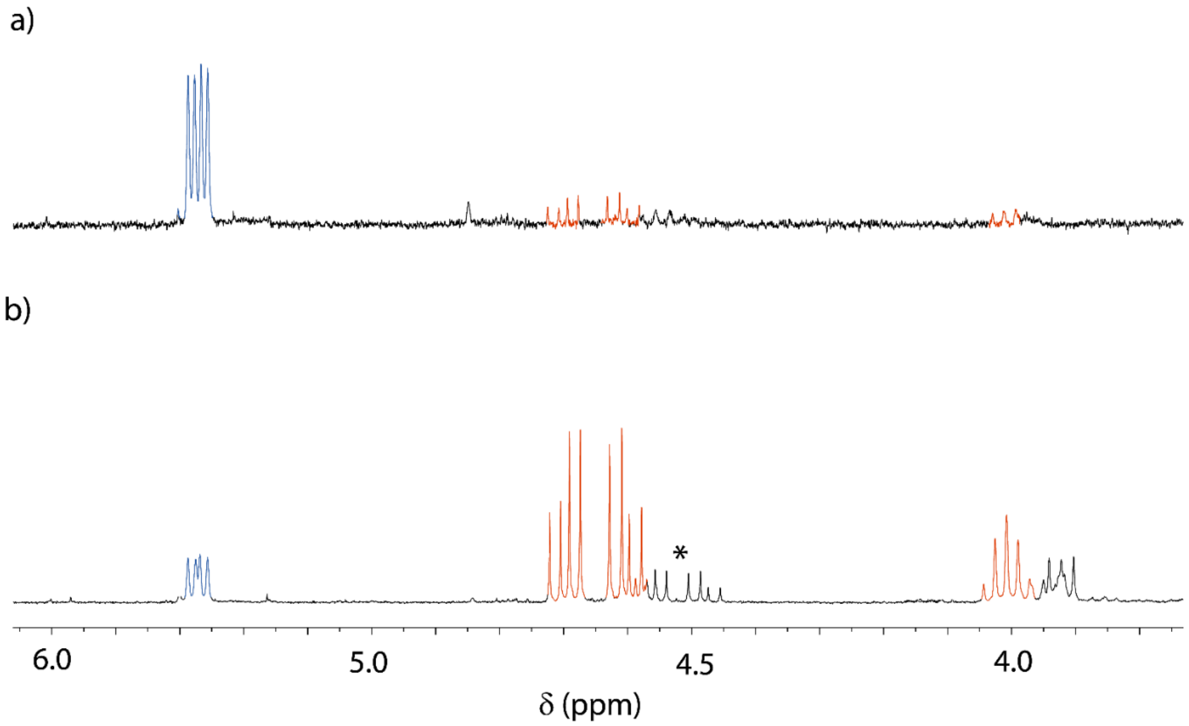

Figure S10. Partial ^1H NMR (400 MHz, $CDCl_3$, 298 K) of the three component reaction crude after 5 days between acetone (1.5 equiv.), *p*-nitrostyrene (1 equiv.) and β-*trans*-nitrostyrene (1 equiv.) in CH_2Cl_2 at room temperature in the presence of 10 mol% of **6b**: a) Without *N*-hexylthymine; b) with *N*-hexylthymine (5 equiv.). In red, signals related to the Michael adduct **9**; In blue, signals related to the aldol adduct **10**. The Michael adduct derived from a double addition of nitrostyrene was also detected (*).

13. Computational studies of the complex 6a·T'

Initial search of conformational space of the computational model **6a·T′** (with **T′** = N^1-methylthymine) were done by using the semiempirical quantum mechanical methods GFN1-xTB and the crest utility program implemented in the xtb program,[1] which was recently developed by S. Grimme.[2] Next, the geometry of the conformer **6a·T′** of lowest energy was re-optimized at M06/cc-pVDZ[3] theoretical level. The minima nature of the computed geometry was confirmed by a frequency analysis at the same computational level. DFT calculations were performed by using the Gaussian 09 software.[4]

---

[1] Grimme, S. *J. Chem. Theory Comput*. 2019, **15**, 2847-2862
[2] Grimme, S.; Bannwarth, C.; Shushkov, P. A J. *Chem. Theory Comput*. 2017, **13**, 1989-2009
[3] a) Zhao, Y.; Truhlar, D. G. *Theor. Chem. Acc*. 2008, **120**, 215; b) Zhao, Y.; Truhlar, D. G. *Acc. Chem. Res*. 2008, **41**, 157; c) Dunning Jr., T. H. *J. Chem. Phys*. 1989, **90**, 1007-1023.
[4] Frisch, M. J.; Trucks, G. W.; Schlegel, H. B.; Scuseria G. E.; et al, Gaussian 09, Revision E.01, Gaussian, Inc., Wallingford CT, 2013.

Cartesian Coordinates of the computed 6a · T

N	-4.145931	-2.274416	-0.318292
C	-3.211943	-2.888675	-1.053623
C	-3.409124	-4.139513	-1.644631
C	-4.642062	-4.744489	-1.427065
C	-5.626414	-4.133968	-0.656068
C	-5.324775	-2.877999	-0.120791
N	-6.207329	-2.111889	0.639119
C	-7.579446	-2.211197	0.647943
O	-8.202137	-3.160145	0.204651
C	-8.277513	-0.984622	1.191400
H	-7.567034	-0.308594	1.693734
H	-9.028684	-1.327688	1.921947
C	-9.003853	-0.251138	0.047509
C	-9.953145	0.815553	0.543933
C	-9.785148	1.468818	1.767187
C	-10.658758	2.481844	2.161608
C	-11.715280	2.857846	1.338611
C	-11.893700	2.212661	0.115603
C	-11.019535	1.204438	-0.274314
H	-11.156937	0.703014	-1.238579
H	-12.722881	2.496055	-0.538243
H	-12.401936	3.649488	1.649414
H	-10.510646	2.978307	3.124532
H	-8.959650	1.190388	2.428630
C	-8.012203	0.288456	-0.976119
C	-7.643815	1.636087	-1.019224
C	-6.706245	2.091036	-1.946674
C	-6.101476	1.203458	-2.831901
C	-6.456022	-0.144270	-2.795700
C	-7.414527	-0.589806	-1.890839
H	-7.707394	-1.645418	-1.890889
H	-5.988629	-0.853775	-3.484238
H	-5.356886	1.552935	-3.553113
H	-6.452650	3.155634	-1.976335
H	-8.112863	2.347883	-0.332436
H	-9.611598	-1.020015	-0.466204
H	-5.806944	-1.236214	1.000101
H	-6.598576	-4.585458	-0.471934
H	-4.842721	-5.724476	-1.868647
H	-2.622048	-4.584531	-2.251834
N	-2.042993	-2.151410	-1.169378
C	-1.023197	-2.268651	-2.073799
O	-0.854218	-3.201832	-2.836220
C	-0.108759	-1.035242	-2.048752
N	-0.176624	-0.369887	-0.750207
H	0.113452	0.605718	-0.862228
C	0.795256	-1.104785	0.048135
H	1.092341	-0.542411	0.946657
H	0.334067	-2.051184	0.387379
C	1.966824	-1.445753	-0.877938
C	1.359135	-1.424089	-2.278502
H	1.878655	-0.665389	-2.885430
H	1.441108	-2.391892	-2.795615
H	2.460074	-2.396268	-0.623993
O	2.972145	-0.398615	-0.808213
C	3.709767	-0.369833	0.298969
O	3.645494	-1.206395	1.184510
C	4.573259	0.855588	0.327389
H	3.881302	1.706370	0.180973
H	5.220064	0.833922	-0.572088
C	5.363668	1.016207	1.622199
C	5.648171	2.465606	1.957199
C	5.778257	3.459353	0.982534
C	6.047614	4.778400	1.345009
C	6.190607	5.126146	2.684731
C	6.069155	4.142121	3.664454
C	5.803060	2.826893	3.300096
H	5.715527	2.052609	4.071282
H	6.181238	4.401708	4.720409
H	6.399937	6.161093	2.966976
H	6.149683	5.538356	0.564579
H	5.682548	3.210237	-0.079945
C	6.632080	0.181217	1.633783
C	6.714198	-0.973823	2.414302
C	7.880238	-1.737813	2.437050
C	8.979760	-1.356290	1.672622
C	8.903855	-0.208735	0.883451
C	7.740014	0.553352	0.865222
H	7.693980	1.453956	0.242530
H	9.761439	0.098688	0.278944
H	9.897065	-1.950401	1.692634
H	7.929220	-2.635112	3.060150
H	5.846739	-1.278300	3.008784
H	4.723778	0.618857	2.430669
H	-0.506276	-0.361511	-2.826484
H	-1.991809	-1.279401	-0.636540
N	5.060362	1.114755	-3.821618
C	5.631679	2.114181	-3.099453
O	6.826862	2.168242	-2.841863
C	4.689602	3.177151	-2.627307
C	3.394653	2.873983	-2.219507
C	2.575131	3.860594	-1.669678
C	3.041956	5.174610	-1.582896
C	4.330731	5.485962	-2.012215
C	5.162993	4.486657	-2.505000
H	6.194110	4.697687	-2.799891
H	4.690096	6.515998	-1.949855
H	2.397552	5.971109	-1.198198
C	1.252188	3.397210	-1.161785
O	0.746551	2.355673	-1.567067
N	0.661523	4.165645	-0.200228
H	1.242147	4.875756	0.233641
C	-0.371369	3.572871	0.625302
C	0.143792	2.569167	1.628566
C	-0.746678	1.626368	2.151613
C	-0.325468	0.687888	3.083119
C	1.008190	0.663956	3.512265
C	1.893891	1.608717	2.995711
C	1.470144	2.551561	2.059413
H	2.199748	3.270316	1.666857
H	2.938482	1.615885	3.328813
C	1.488350	-0.445440	4.409947
N	1.620558	-1.684195	3.660950
C	0.544901	-2.532219	3.549069
O	-0.468057	-2.412977	4.221429
C	0.686269	-3.579339	2.492873
C	1.926863	-3.929193	1.964082
C	2.020379	-4.760428	0.849744
C	0.853631	-5.295594	0.296668
C	-0.386877	-4.994653	0.854197
C	-0.473608	-4.137254	1.948403
H	-1.437222	-3.858643	2.382953

H	-1.296883	-5.417148	0.420094
H	0.946183	-5.943845	-0.577915
C	3.328992	-5.057538	0.186957
O	3.463633	-5.988547	-0.592362
N	4.340936	-4.188591	0.501972
H	4.093724	-3.287316	0.909774
C	5.609204	-4.234279	-0.199267
C	5.690972	-3.166841	-1.257232
C	6.646195	-2.152724	-1.190765
C	6.694157	-1.157301	-2.164014
C	5.781185	-1.157573	-3.219662
C	4.817346	-2.168864	-3.281293
C	4.771858	-3.164334	-2.312906
H	4.027945	-3.966814	-2.374635
H	4.103156	-2.186106	-4.112651
C	5.814830	-0.056588	-4.243276
H	6.848150	0.278984	-4.417335
H	5.401498	-0.402326	-5.203663
H	7.445084	-0.361763	-2.106903
H	7.365722	-2.139950	-0.364700
H	6.437391	-4.118947	0.519944
H	5.679704	-5.238045	-0.646572
H	2.836642	-3.549032	2.434624
H	2.275635	-1.650975	2.879628
H	2.462684	-0.188896	4.857444
H	0.772960	-0.637394	5.222783
H	-1.020929	-0.062125	3.472109
H	-1.786154	1.621201	1.802089
H	-1.090060	3.066523	-0.036985
H	-0.917457	4.387392	1.132245
H	3.009165	1.849437	-2.241752
H	4.055690	1.118054	-3.955852
C	-2.549536	3.379240	-2.242783
N	-3.251807	2.669695	-1.184647
C	-3.904497	3.331260	-0.170447
C	-4.627447	2.712198	0.790568
C	-4.722440	1.259774	0.733359
O	-5.389831	0.586118	1.514244
N	-3.986401	0.658166	-0.271954
C	-3.300230	1.280444	-1.291914
O	-2.772695	0.685500	-2.210414
H	-4.052793	-0.379013	-0.330902
C	-5.367157	3.419309	1.872474
H	-5.210626	4.506947	1.825915
H	-5.055510	3.058214	2.865698
H	-6.449241	3.217460	1.799010
H	-3.808920	4.421997	-0.195551
H	-3.114670	3.339900	-3.187844
H	-2.418236	4.427159	-1.938978
H	-1.565410	2.915892	-2.406137

14. DOSY NMR experiments for *N*-hexylthymine and rotaxane 6b

In order to prove the association between our DAP-based systems and *N*-hexylthymine (**T**) we performed ^{1}H PGSE (Pulsed Gradient Spin Echo) diffusion measurements on solutions of **T** and rotaxane **6b**, and a mixture of both of them (CD_2Cl_2, 298K). Having in mind the low association constant calculated for **6b** and **T**, we prepared three different samples: a) **6b** (2 mM); b) **T** (2 mM); and c) **6b** (2 mM) + **T** (0.26 mM). The obtained D values are despite in Table S7.

The variation of the D values of **T** when is uncomplexed (15.86 x 10^{10}) and in the presence of excess of rotaxane **6b** (14.52 x 10^{10}) was calculated to be a 8.2 % further proving the formation of the complex **6b·T** (Table S8) in these measurement conditions.

Table S7. Diffusion coefficients (D [$m^2 s^{-1}$]) measured in CD_2Cl_2 (2 mM) at 298 K[a], hydrodynamic radii (r_H [Å]) of **6b**, **T** and **6b·T complex**.

Entry	Compound	D x 10^{10} [b]	r_H(PGSE)[c,d]	r_H(model)
1	**T**	15.86[e]	4.5[f]	4.6
2	**6b**	6.92[g]	8.2	8.1
3	**6b·T**	6.77[g] (14.52[e])	8.4 (4.9)[f]	8.5

[a] η (CD_2Cl_2, 298 K) = $0.38\cdot10^{-3}$ Kg s^{-1} m^{-1};[5] [b] Experimental error is *ca.* ± 2%; [c] Calculated from the D values by using the Stokes-Einstein equation; [d] Standard deviation is approximately ± 0.1 Å; [e] Measured at 3.67 ppm (**T**); [f] Calculated from the D value by using the Stokes-Einstein equation, with the correction proposed by Chen;[6][g] Measured at 2.97 ppm (**6b**).

[5] Aminabhavi, T. M., Banerjee, K. *J. Chem. Eng. Data*, 1998, *43*, 1096-1101
[6] Chen, H.-C.; Chen, S.-H. *AlChE J.* 1985, **31**, 76-81

Table S8. Variation of the diffusion coefficients (D [m^2 s^{-1}]) measured in CD_2Cl_2 (2 mM) at 298 K[a] between **6b** and **T** and **6b·T** complex.

	D x 10^{10}	
	**T**	**6b**
Free Compound	15.86	6.92
Complex	14.52	6.77
ΔD (%)	**8.2 %**	**2.2 %**

Experimental procedure:

The PGSE NMR diffusion measurements were performed on a 600 MHz Bruker AVANCE spectrometer, equipped with a microprocessor-controlled gradient unit and a multinuclear inverse probe with an actively shielded Z-gradient coil. The sample was not spun and the airflow was disconnected. The shape of the gradient pulse was rectangular, and its strength varied automatically during the course of the experiments. The D-values were determined from the slope of the regression line ln(I/I_o) vs G^2, according to the erquation:

$$\ln(I/I_o) = -(\gamma\delta)^2 G^2(\Delta-\delta/3)D$$

I/I_o = observed spin echo intensity/intensity without gradients, G = gradient strength, Δ = delay between the midpoints of the gradients, D = diffusion coefficient, δ = gradient length.

The Δ value was set at 150 ms. All the measurements were carried out using the ^{1}H resonances. The gradient length was set in the range of 1.0 and 2.0 ms. D1 was set to $5T_1$. The number of scans was 32. All of the observed data leading to the reported D-values afforded lines whose correlation coefficients were above 0.999.

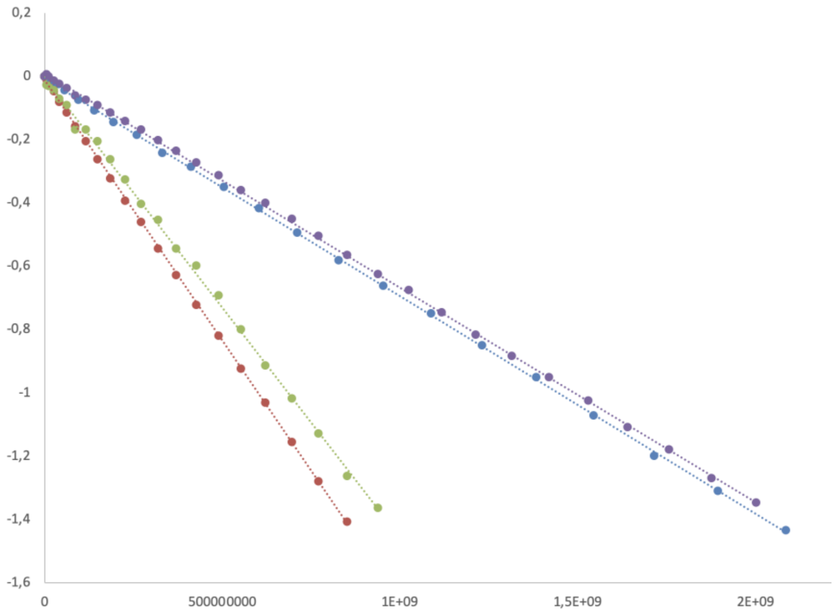

Figure 11. ^{1}H PGSE diffusion experiments (600 MHz, 298 K, 2 mM in CD_2Cl_2) for N-hexylthymine (**T**) (●), **6b·T** [δ (**T**)= 3.67 ppm] (●), **6b** (●), **6b·T** [δ (**6b**)= 2.97 ppm] (●). Plots of the observed intensity changes ln(I/I_o) as a function of $\gamma^2\delta^2(\Delta-\delta/3)G^2$ showing the different translation rates depending on their molecular sizes. The solid lines represent linear least-squares fits to the experimental data. All correlation coefficients were above 0.999.

15. ^{1}H and ^{13}C NMR Spectra of synthesized compounds

2 (^{1}H NMR, 400 MHz, $CDCl_3$, 318 K)

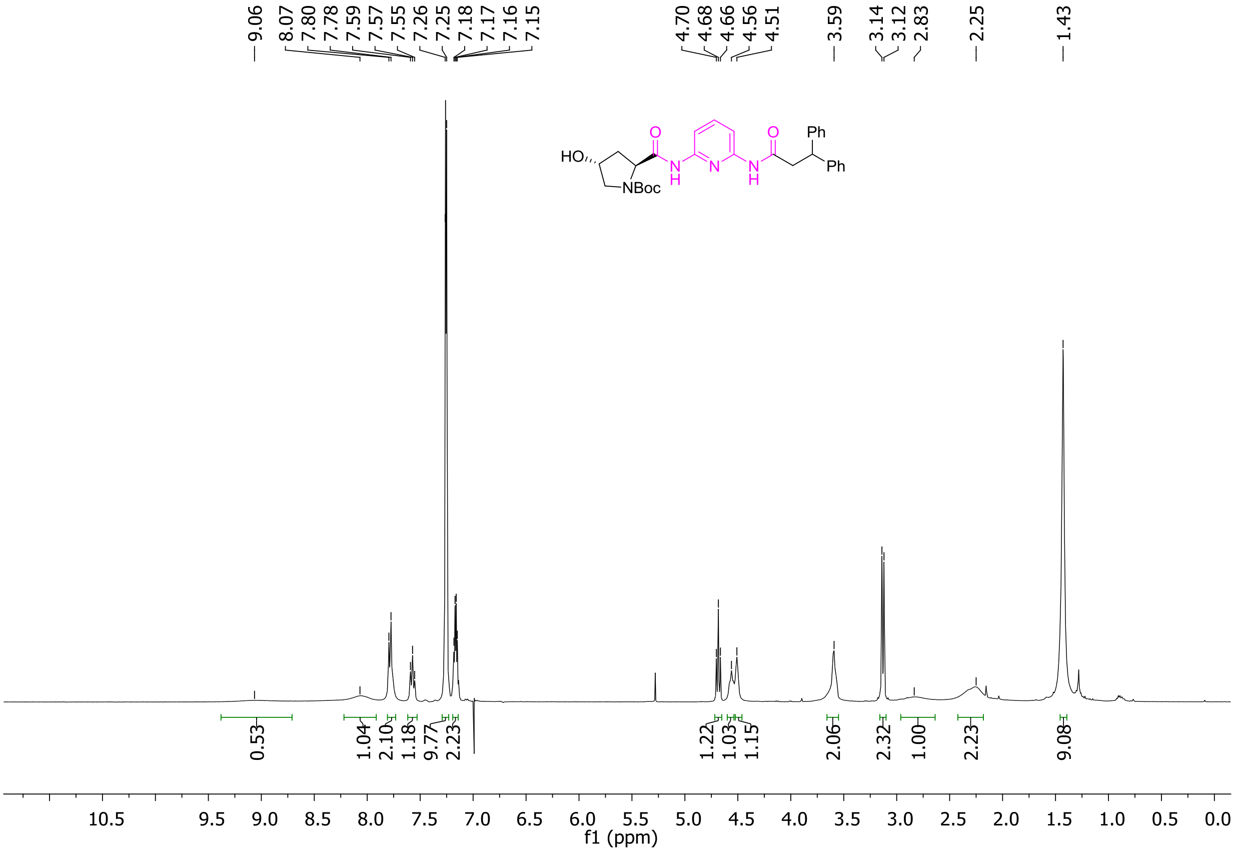

2 (^{13}C NMR, 100 MHz, $CDCl_3$, 318 K)

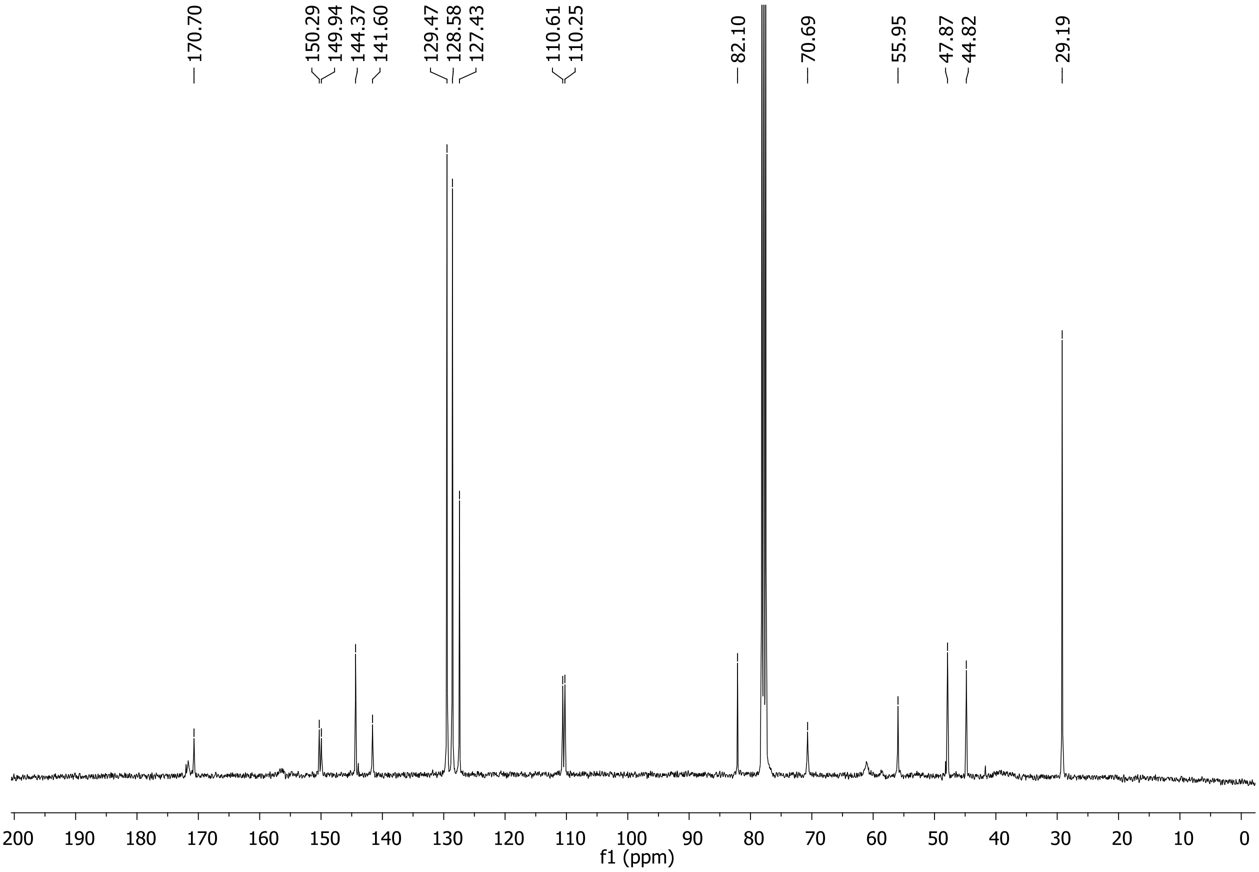

3 (^{1}H NMR, 400 MHz, $CDCl_3$, 318 K)

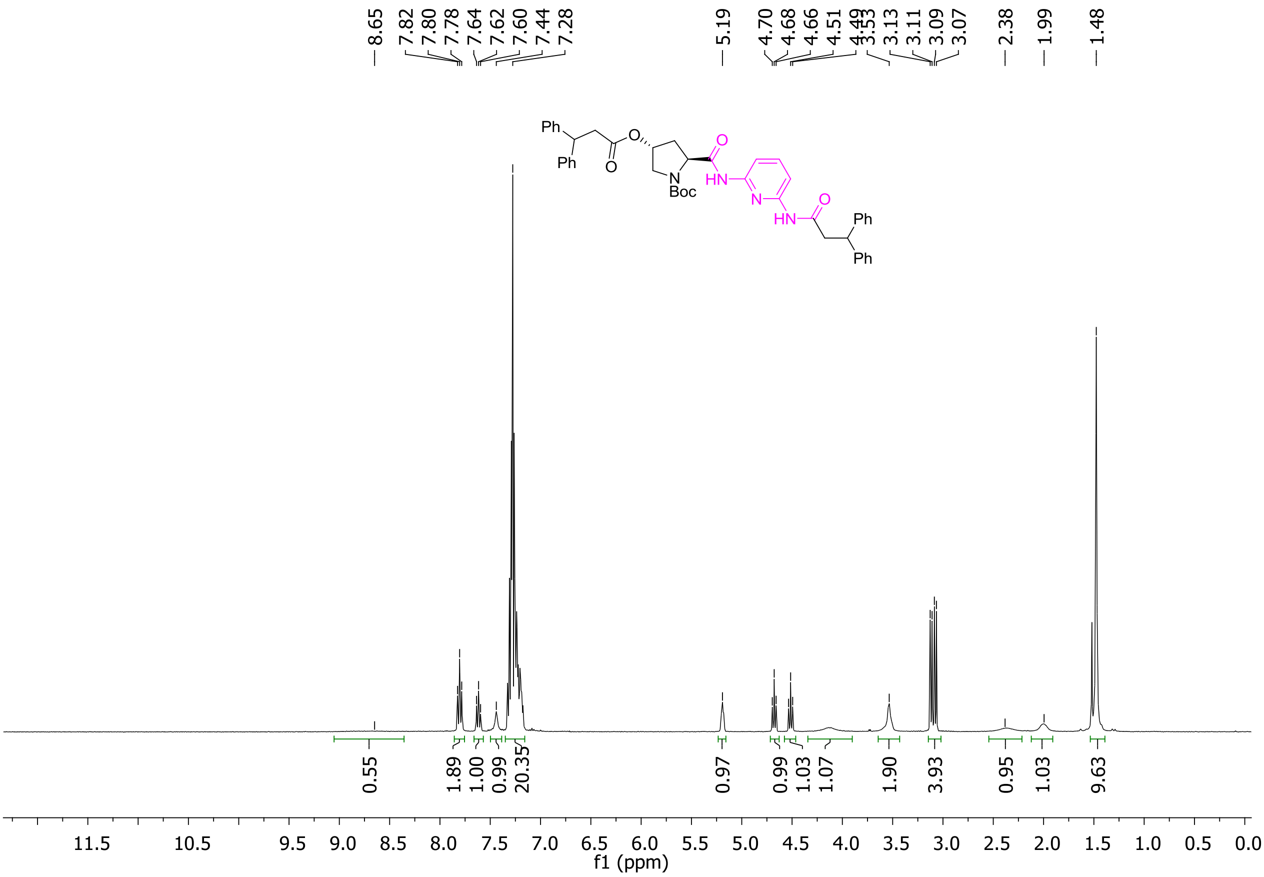

3 (^{13}C NMR, 100 MHz, $CDCl_3$, 318 K)

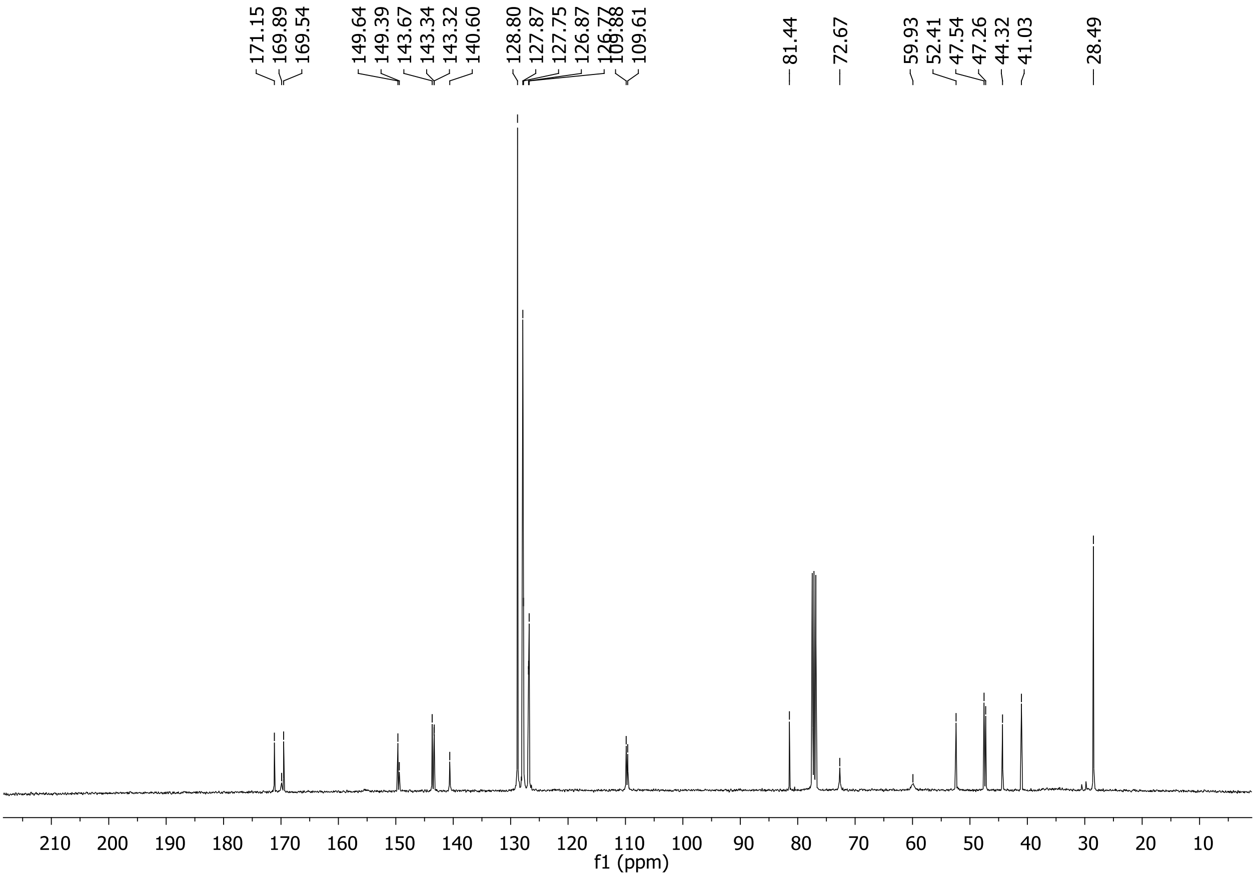

5a (^{1}H NMR, 400 MHz, $CDCl_3$, 318 K)

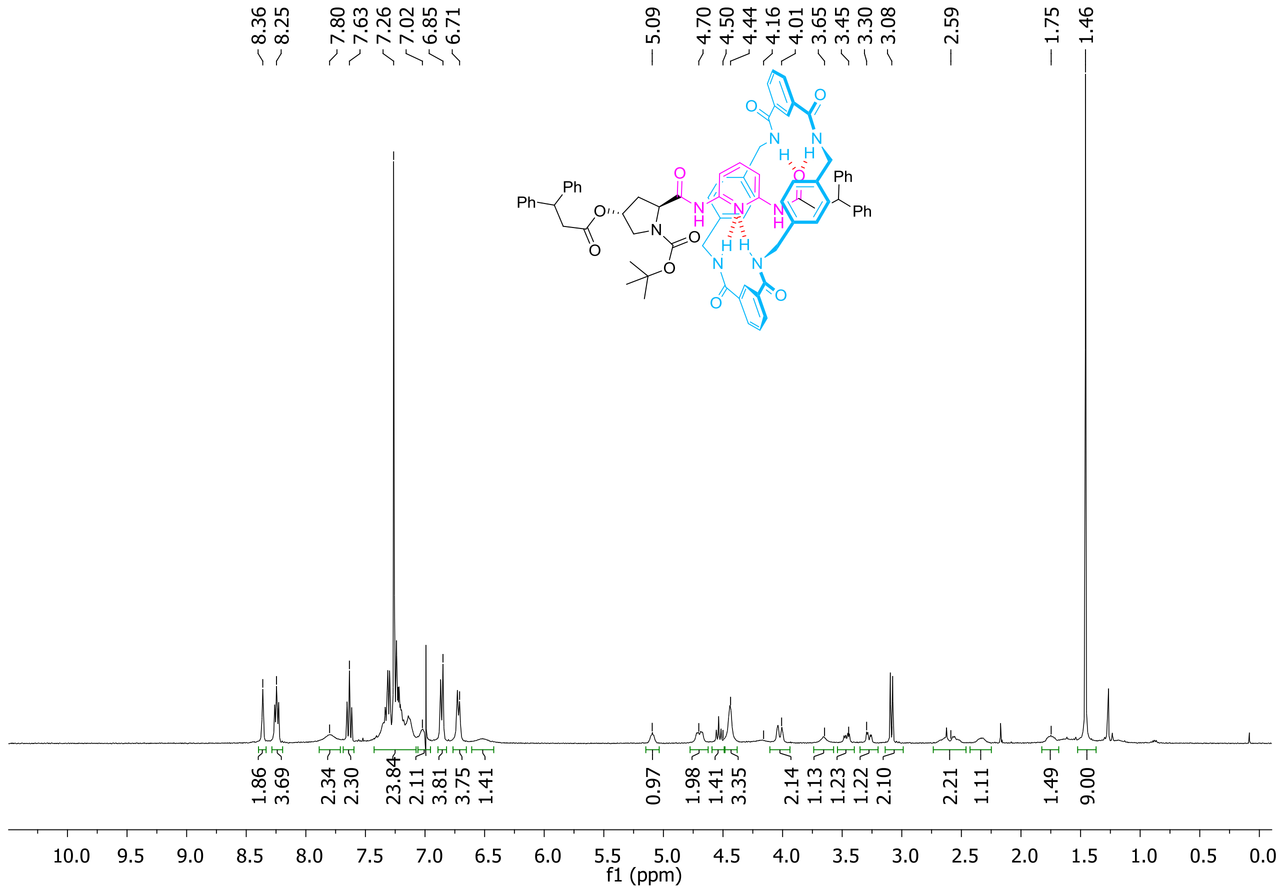

5a (^{13}C NMR, 100 MHz, $CDCl_3$, 318 K)

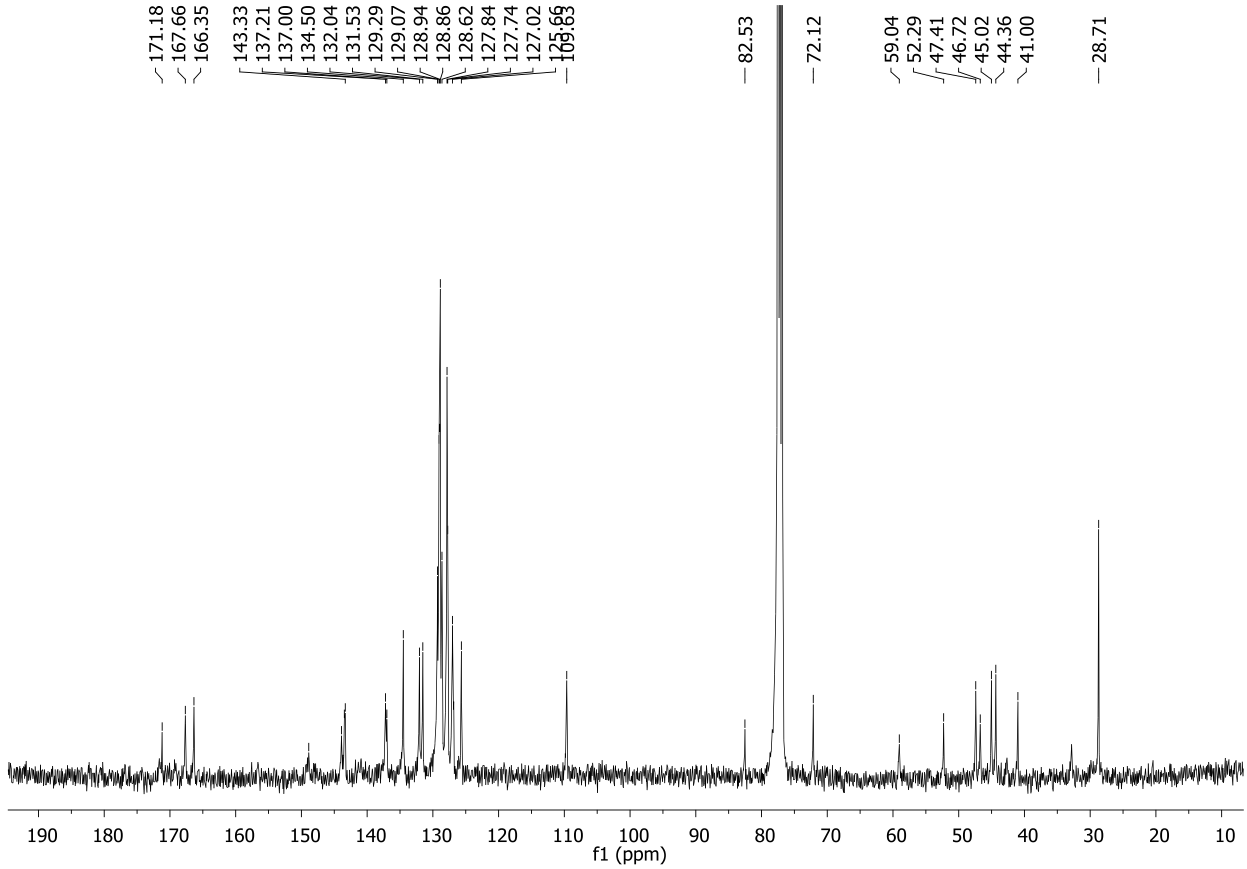

5b (^{1}H NMR, 400 MHz, CDCl$_3$, 318 K)

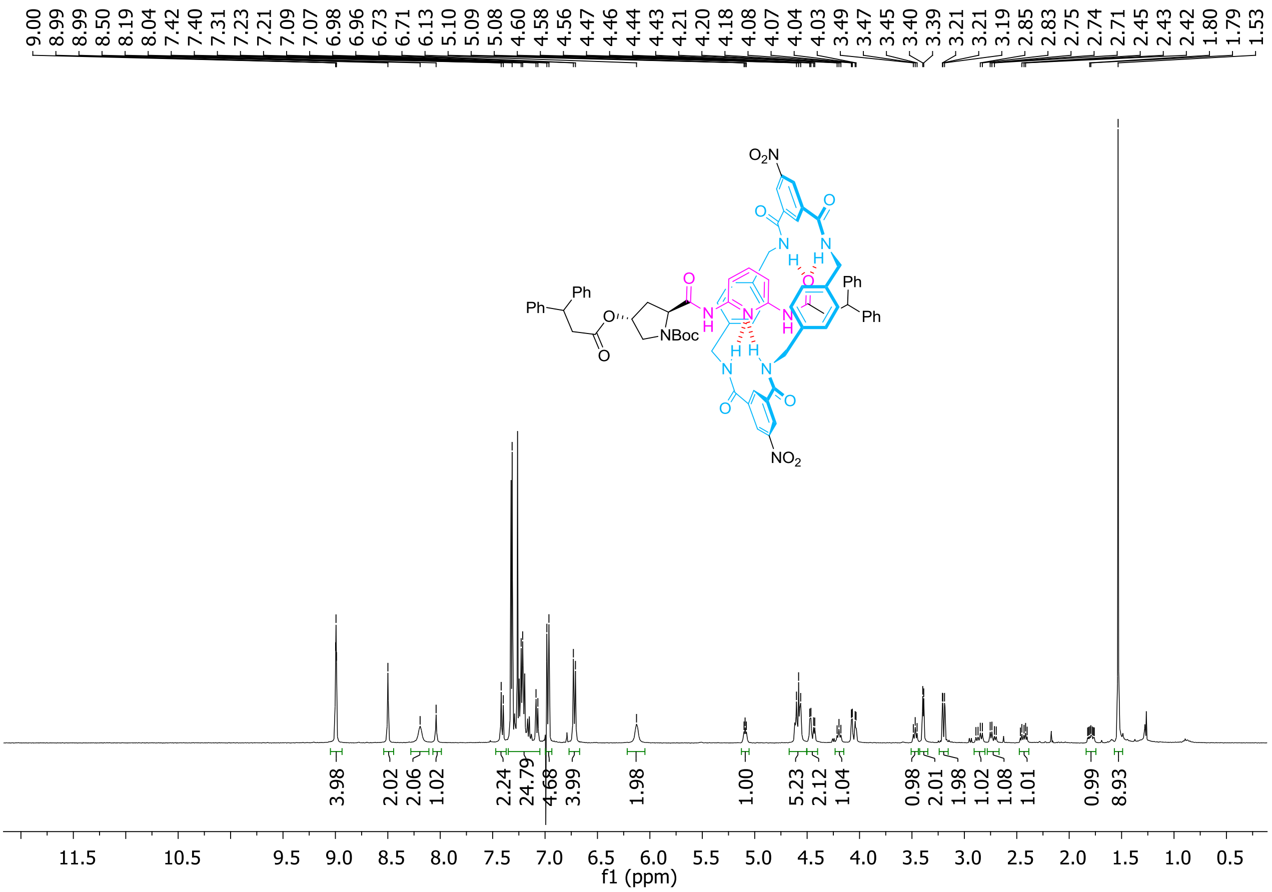

5b (^{13}C NMR, 100 MHz, CDCl$_3$, 318 K)

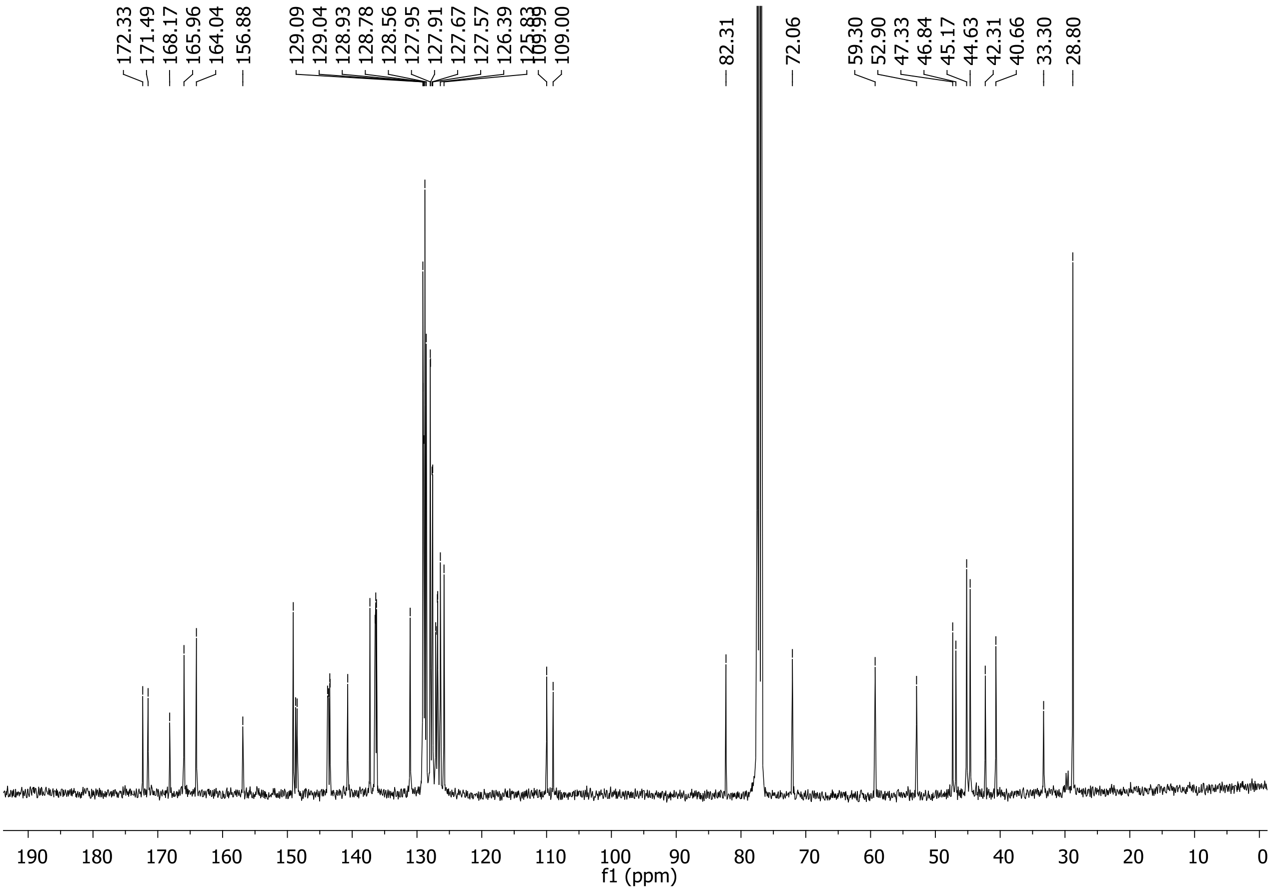

4 (^{1}H NMR, 300 MHz, $CDCl_3$, 298 K)

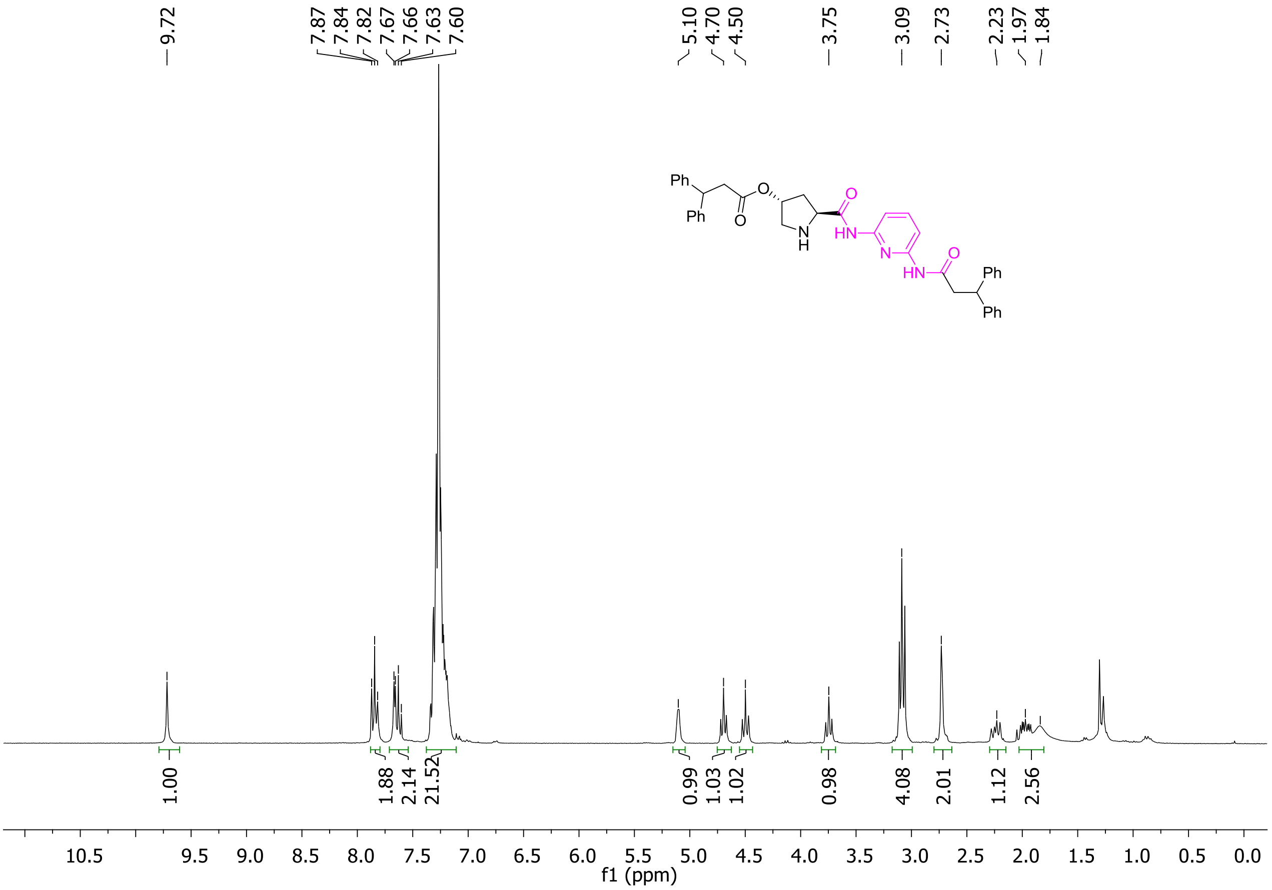

4 (^{1}H NMR, 75 MHz, $CDCl_3$, 298 K)

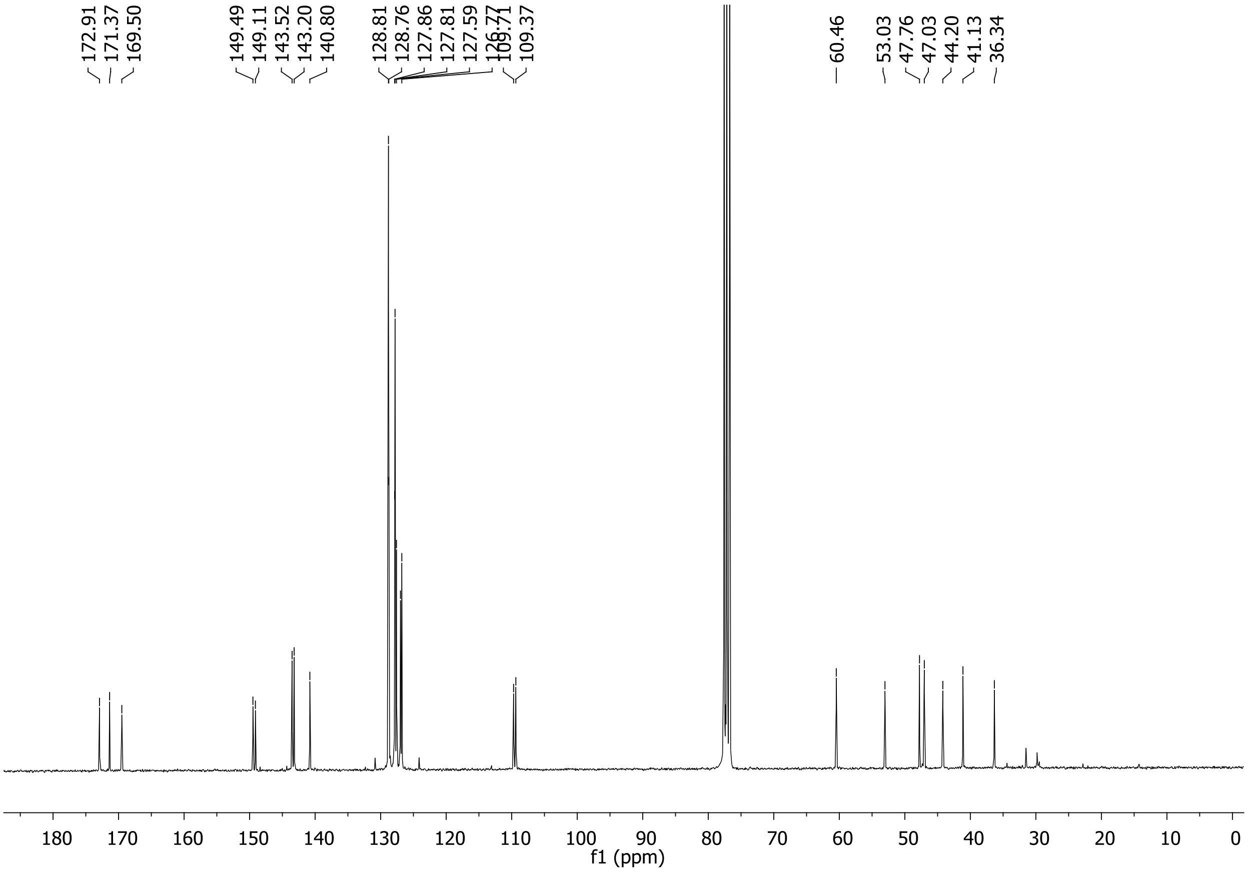

6a (^{1}H NMR, 300 MHz, $CDCl_3$, 298 K)

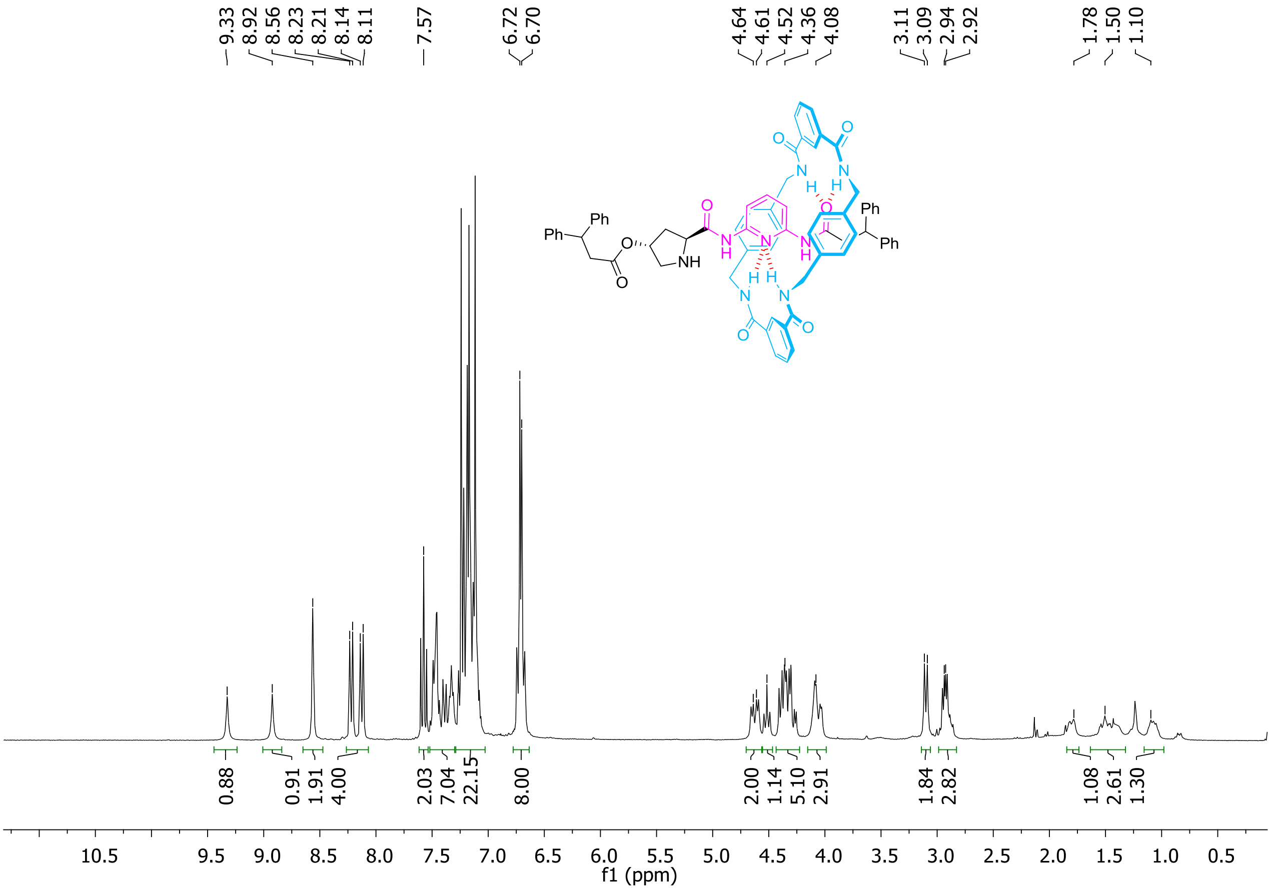

6a (^{13}C NMR, 75 MHz, $CDCl_3$, 298 K)

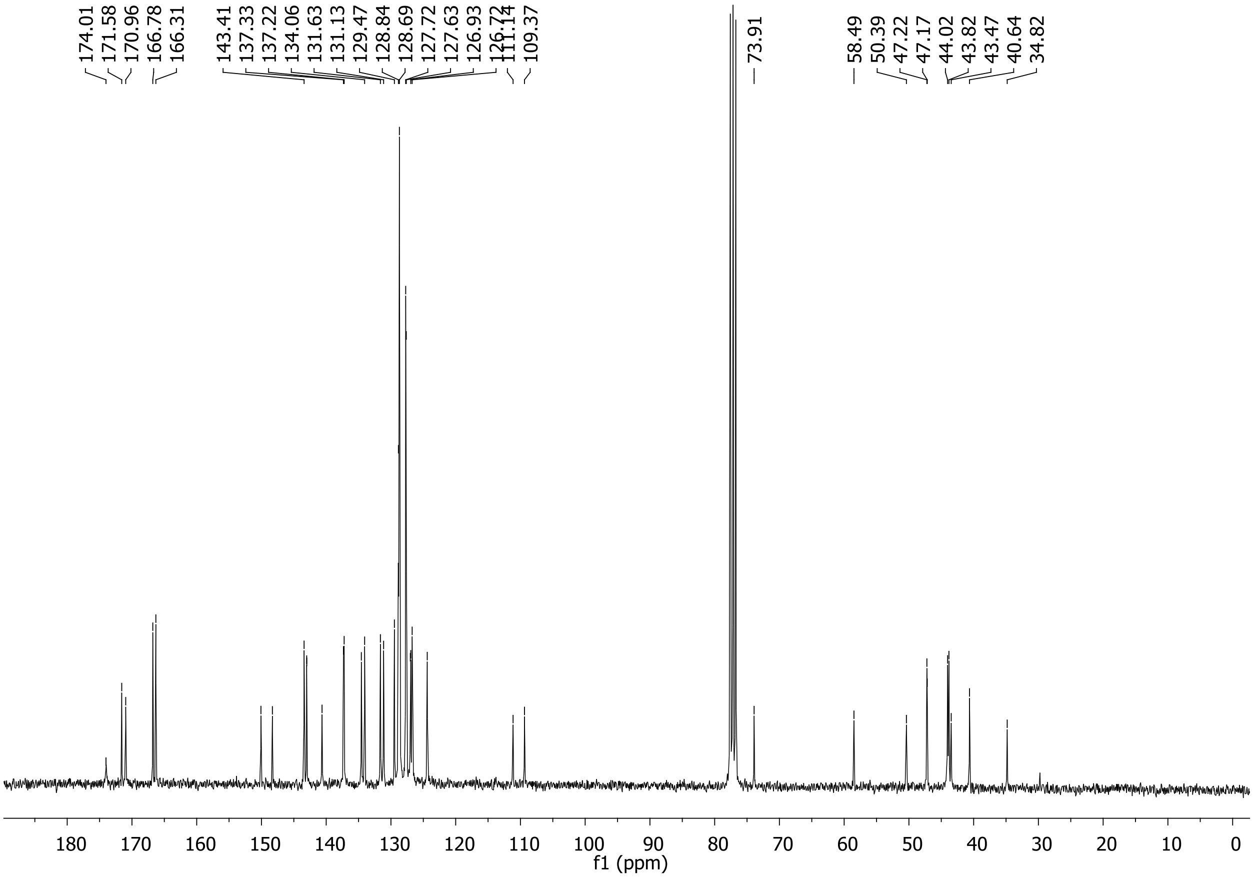

6a (HMQC, $CDCl_3$, 298 K)

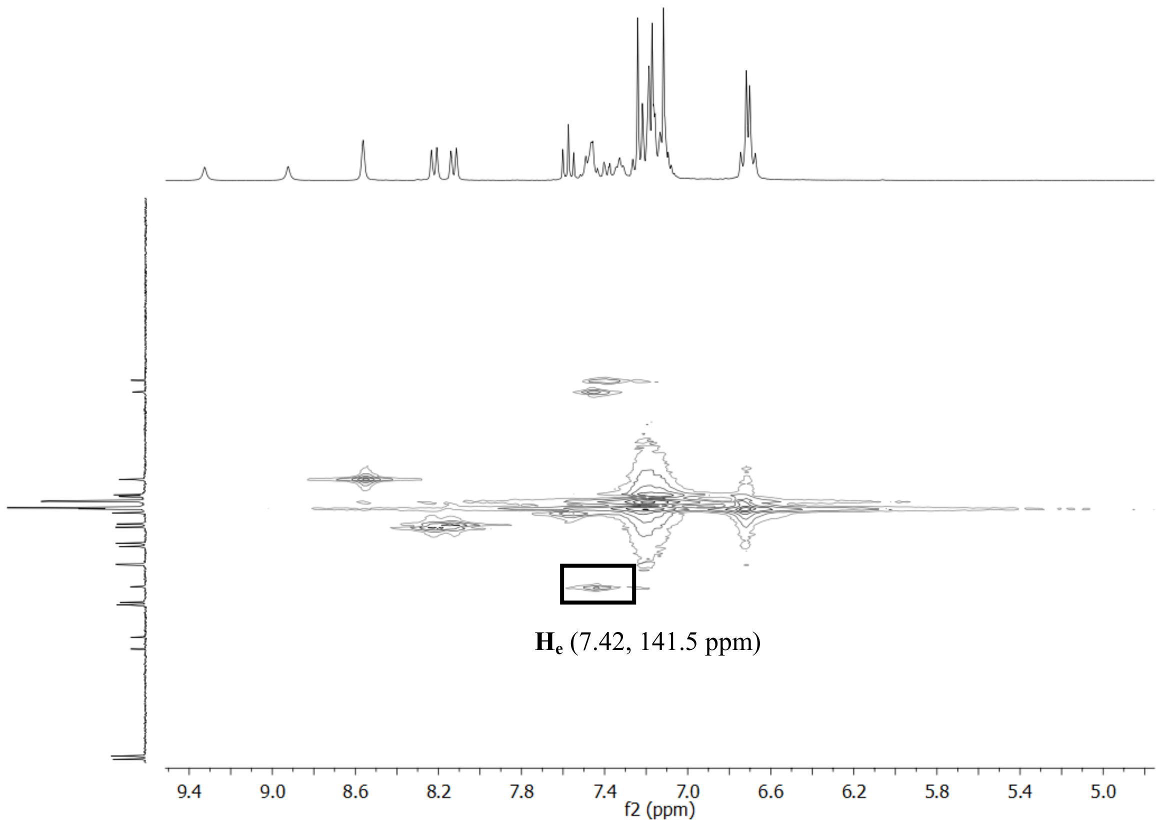

6b (^{1}H NMR, 400 MHz, CD_2Cl_2, 298 K)

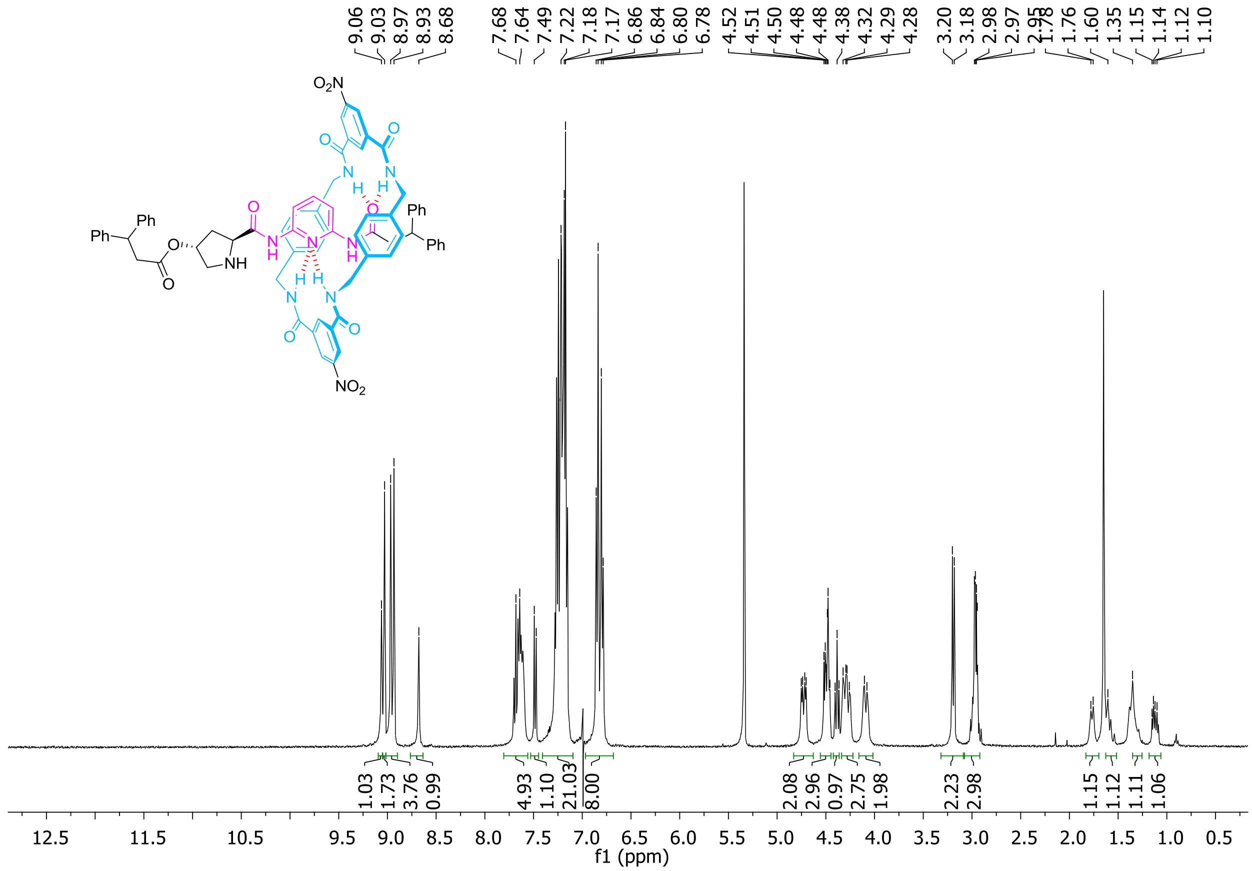

6b (^{13}C NMR, 100 MHz, CD_2Cl_2, 298 K)

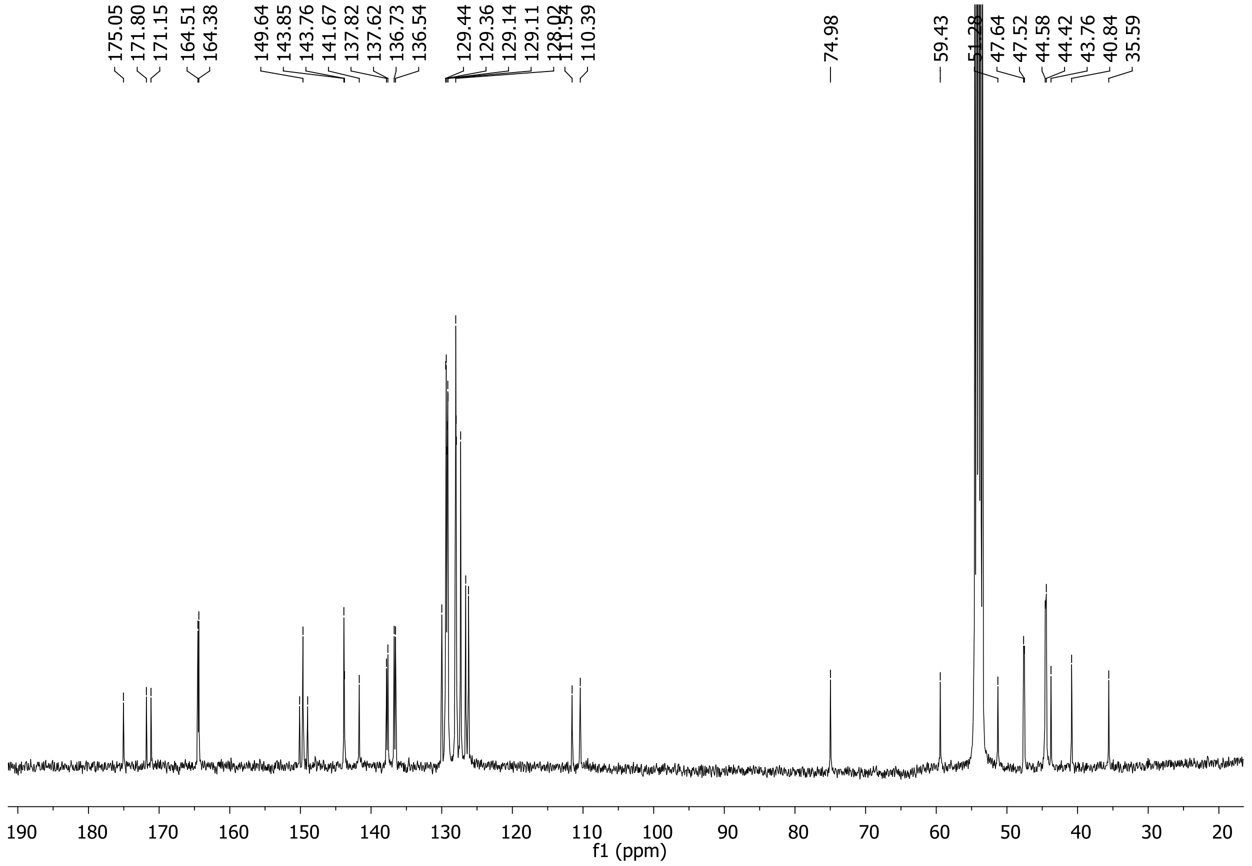

13 (^{1}H NMR, 400 MHz, $CDCl_3$, 298 K)

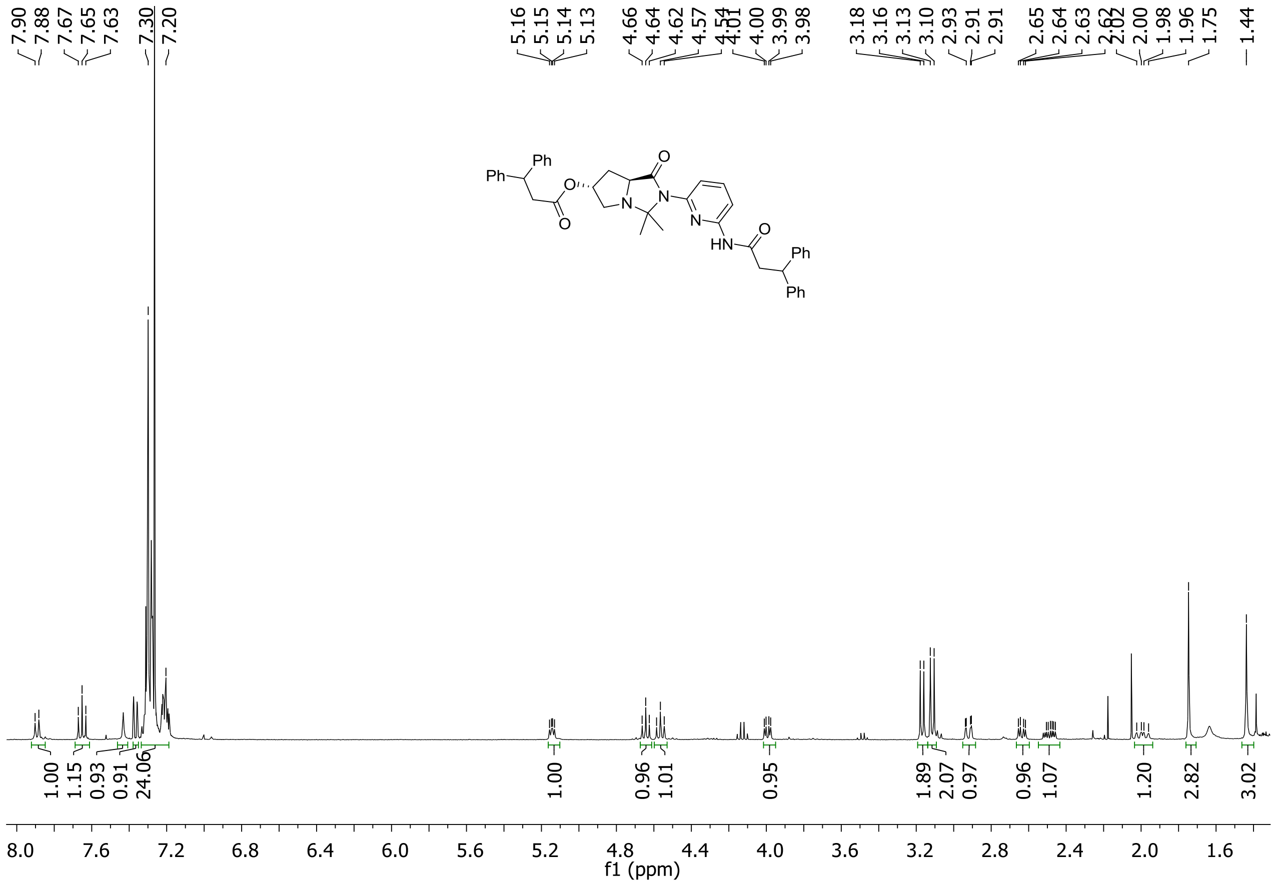

16. Copies of HPLC Traces of Synthesized Compounds

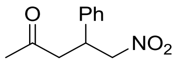

9

Racemate

Peak #	RetTime [min]	Type	Width [min]	Area [mAU*s]	Height [mAU]	Area %
1	10.528	MM	0.3121	582.36694	31.10250	51.2757
2	12.626	MM	0.4934	553.38940	18.69262	48.7243

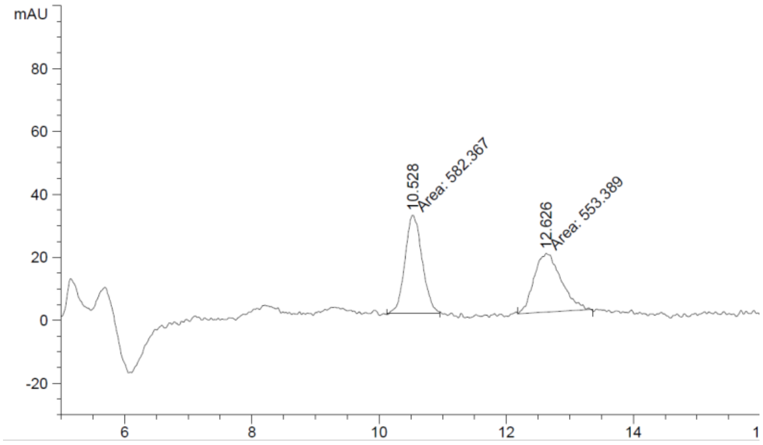

Employing rotaxane 6a as catalyst

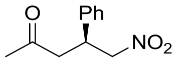

(*S*)-9

Peak #	RetTime [min]	Type	Width [min]	Area [mAU*s]	Height [mAU]	Area %
1	11.148	VV	0.2864	1204.74402	51.59929	57.1834
2	13.436	VV	0.4495	902.06152	24.50077	42.8166

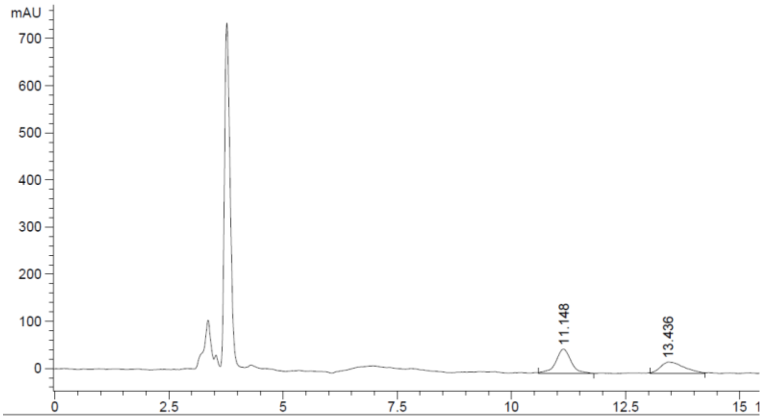

Employing rotaxane 6b as catalyst

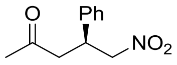

(*S*)-9

Peak #	RetTime [min]	Type	Width [min]	Area [mAU*s]	Height [mAU]	Area %
1	9.253	MM	0.2665	1142.31702	71.42821	54.3852
2	10.832	MM	0.3982	958.10181	40.09809	45.6148

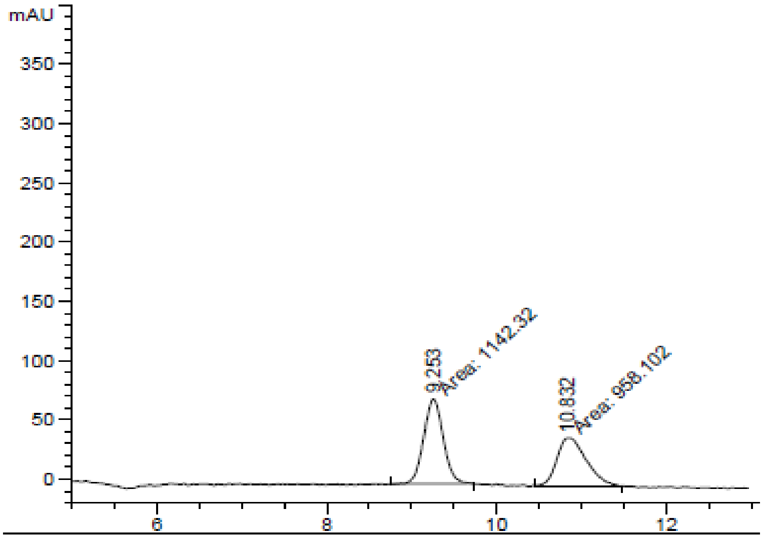

Employing thread 4 as catalyst + 5 equiv. *N*-hexylthymine

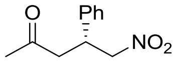

(*R*)-9

Peak #	RetTime [min]	Type	Width [min]	Area [mAU*s]	Height [mAU]	Area %
1	9.925	VV	0.1781	81.5216	7.62504	43.0166
2	12.166	VV	0.2302	110.0510	7.96812	56.9834

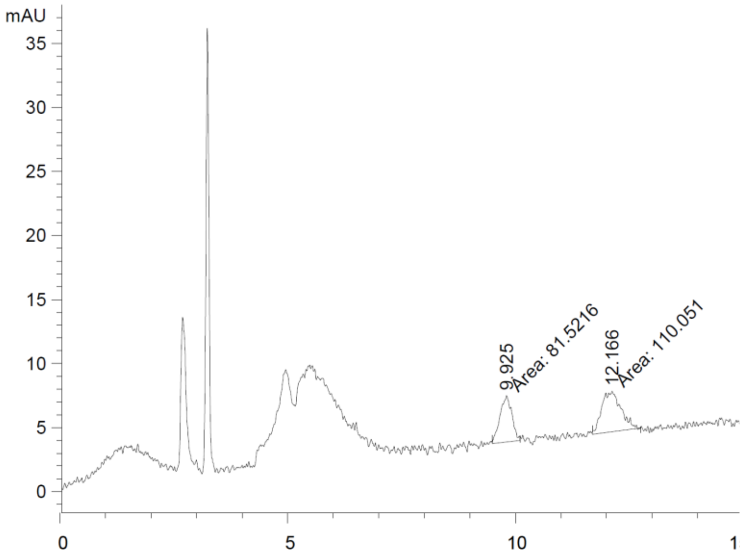

Employing rotaxane 6a as catalyst + 5 equiv. *N*-hexylthymine

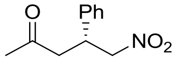

(*R*)-9

Peak #	RetTime [min]	Type	Width [min]	Area [mAU*s]	Height [mAU]	Area %
1	9.229	VV	0.2556	2168.65283	130.65559	23.0028
2	10.598	VV	0.4394	7259.13574	230.52879	76.9972

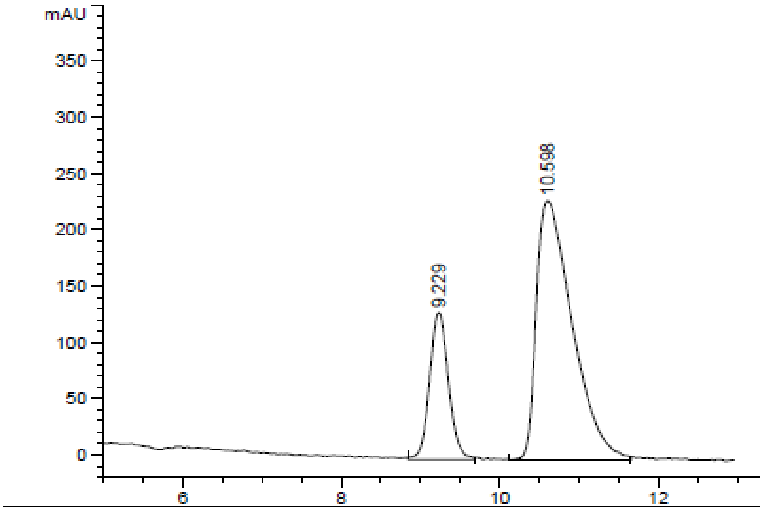

Employing rotaxane 6b as catalyst + 5 equiv. *N*-hexylthymine

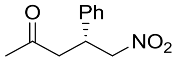

(*R*)-9

Peak #	RetTime [min]	Type	Width [min]	Area [mAU*s]	Height [mAU]	Area %
1	9.235	MM	0.2511	33.64297	2.23331	9.3061
2	10.592	MM	0.5546	327.87332	9.85334	90.6939

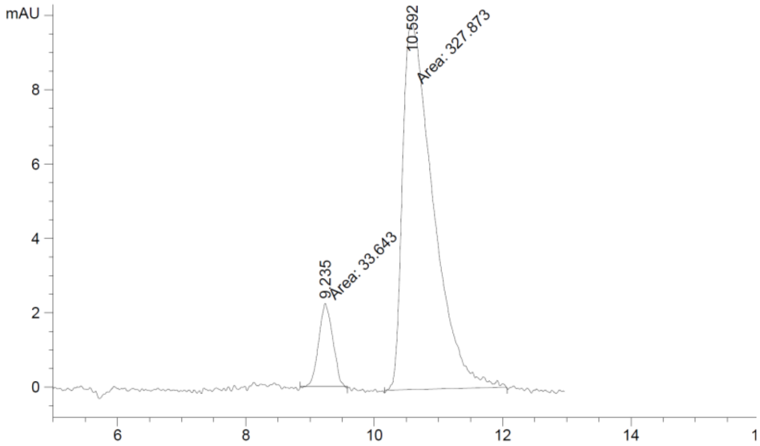

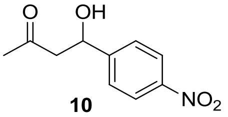

10

Racemate

Peak #	RetTime [min]	Type	Width [min]	Area [mAU*s]	Height [mAU]	Area %
1	8.161	VB	0.3642	2.95848e4	1197.45203	49.1722
2	10.314	VB	0.5646	3.05809e4	824.79163	50.8278

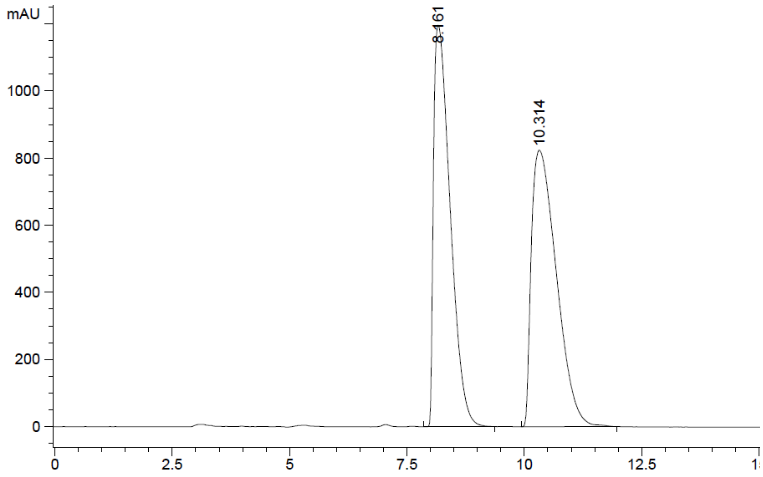

Employing thread 4 as catalyst

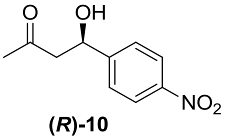

(*R*)-10

Peak #	RetTime [min]	Type	Width [min]	Area [mAU*s]	Height [mAU]	Area %
1	8.310	MM	0.2482	441.63876	29.65220	75.6291
2	10.114	MM	0.3180	142.31482	7.45971	24.3709

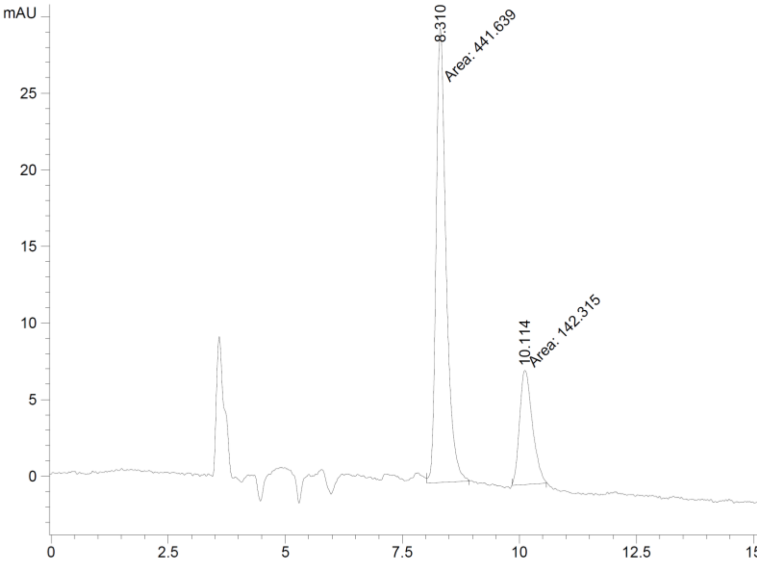

Employing rotaxane 6a as catalyst

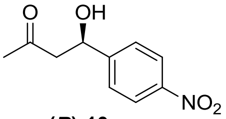

(*R*)-10

Peak #	RetTime [min]	Type	Width [min]	Area [mAU*s]	Height [mAU]	Area %
1	8.804	MM	0.3694	5439.95996	245.44302	77.5820
2	11.019	MM	0.4437	1571.92505	59.04602	22.4180

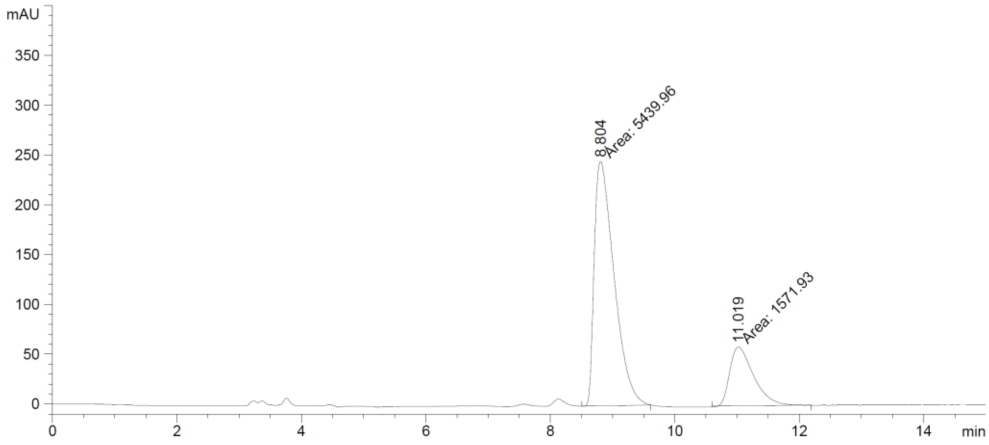

Employing rotaxane 6b as catalyst

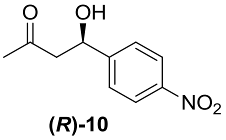

(*R*)-10

Peak #	RetTime [min]	Type	Width [min]	Area [mAU*s]	Height [mAU]	Area %
1	8.979	MM	0.4205	8333.62793	330.33075	87.9469
2	11.393	MM	0.4619	1142.11743	41.21165	12.0531

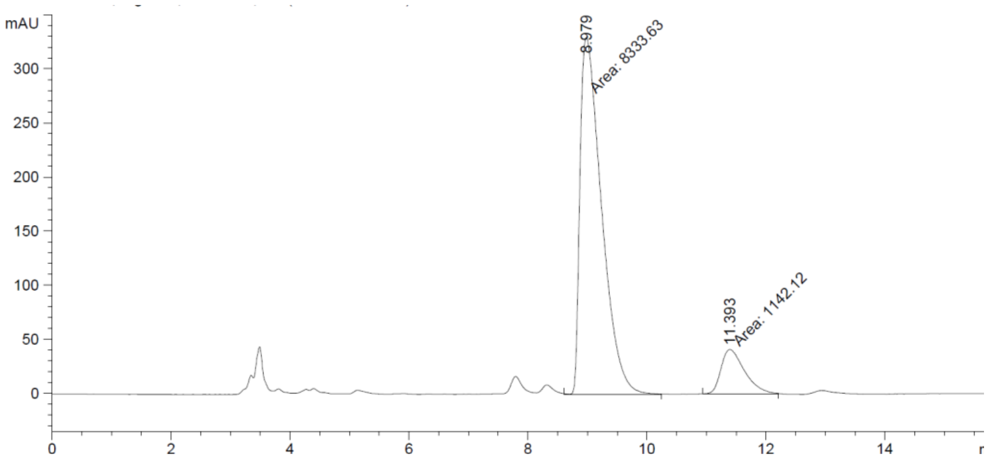

Employing thread 4 as catalyst + 5 equiv. of *N*-hexylthymine

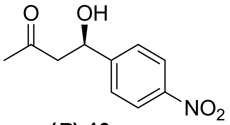

(*R*)-10

Peak #	RetTime [min]	Type	Width [min]	Area [mAU*s]	Height [mAU]	Area %
1	8.800	VB	0.3304	4454.72705	207.51854	62.5249
2	10.941	PB	0.4157	2669.99829	98.03532	37.4751

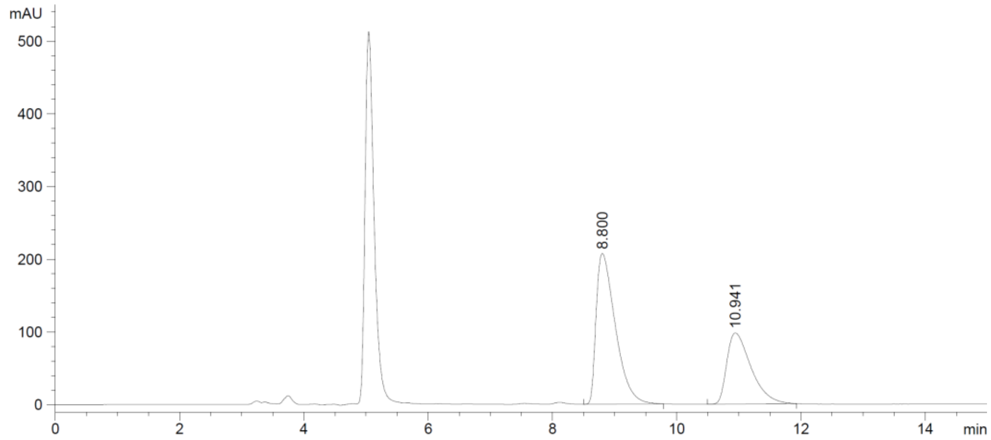

Employing rotaxane 6b as catalyst + 5 equiv. of *N*-hexylthymine

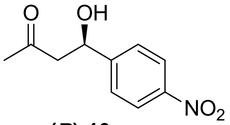

(*R*)-10

Peak #	RetTime [min]	Type	Width [min]	Area [mAU*s]	Height [mAU]	Area %
1	7.986	VB	0.2341	3662.24951	237.23691	71.6526
2	9.692	BB	0.2991	1448.86914	73.14611	28.3474

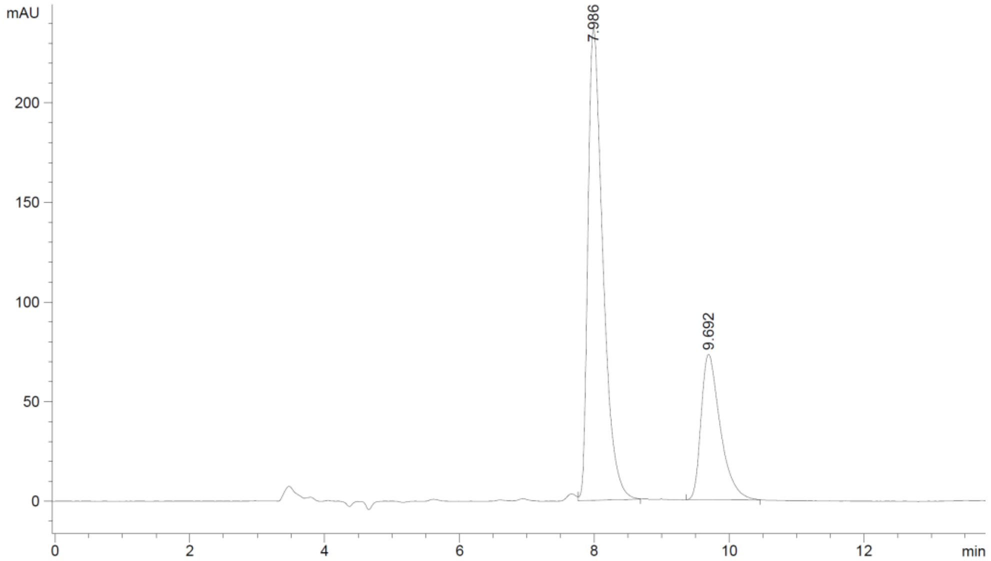

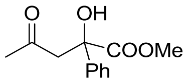

12Me

Racemate

Peak #	RetTime [min]	Type	Width [min]	Area [mAU*s]	Height [mAU]	Area %
1	5.523	MF	0.1643	5591.85791	567.25525	48.1293
2	5.886	FM	0.1993	6026.56104	503.90375	51.8707

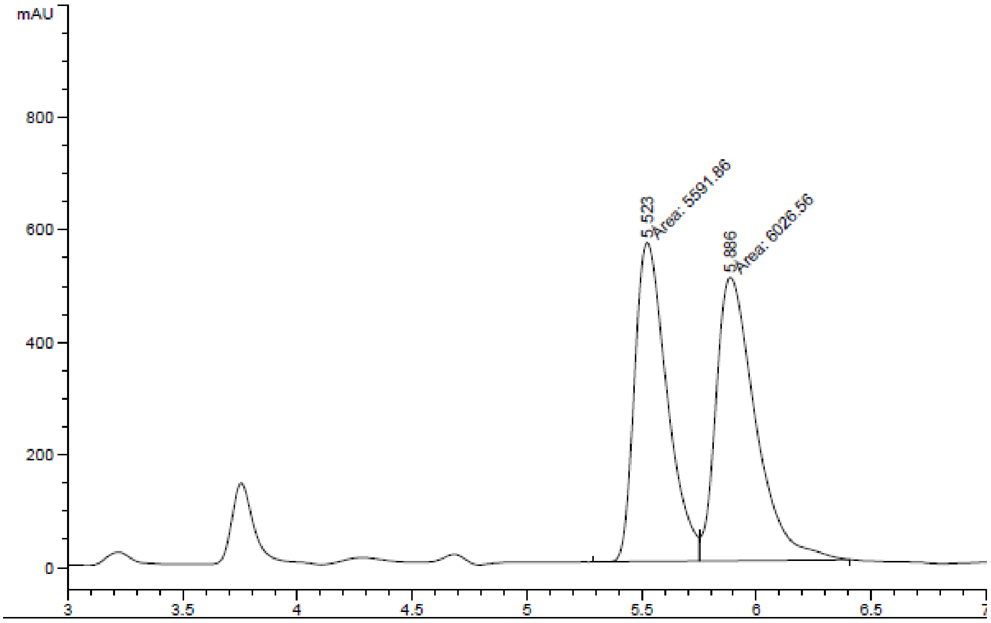

Employing thread 4 as catalyst

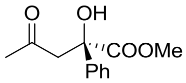

(*R*)-12Me

Peak #	RetTime [min]	Type	Width [min]	Area [mAU*s]	Height [mAU]	Area %
1	5.559	BV	0.1391	532.43146	57.99928	25.2364
2	5.930	VB	0.1652	1577.34753	144.61880	74.7636

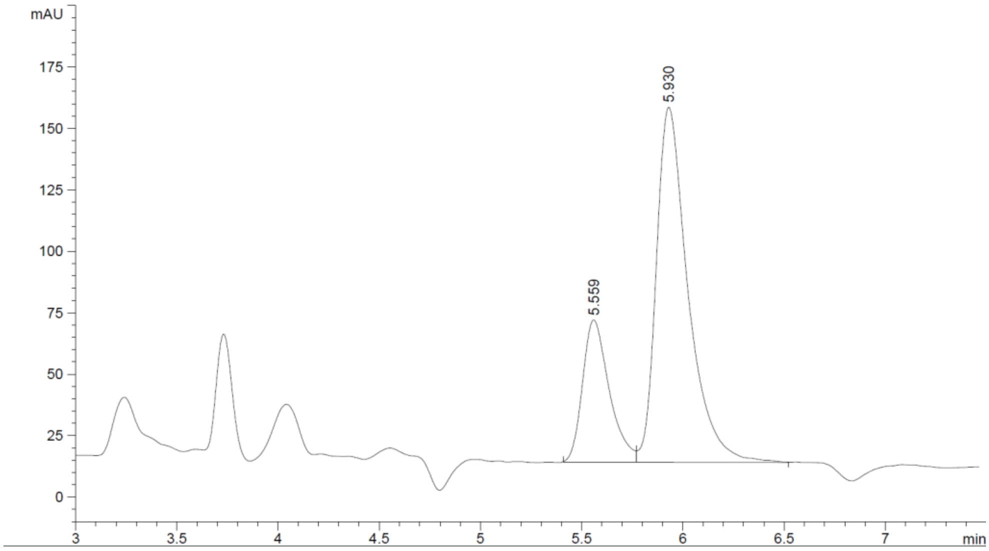

Employing rotaxane 6a as catalyst

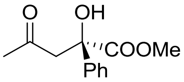

(*R*)-12Me

Peak #	RetTime [min]	Type	Width [min]	Area [mAU*s]	Height [mAU]	Area %
1	5.556	MF	0.1503	129.42201	14.34866	10.9397
2	5.931	FM	0.1738	1053.62170	101.01386	89.0603

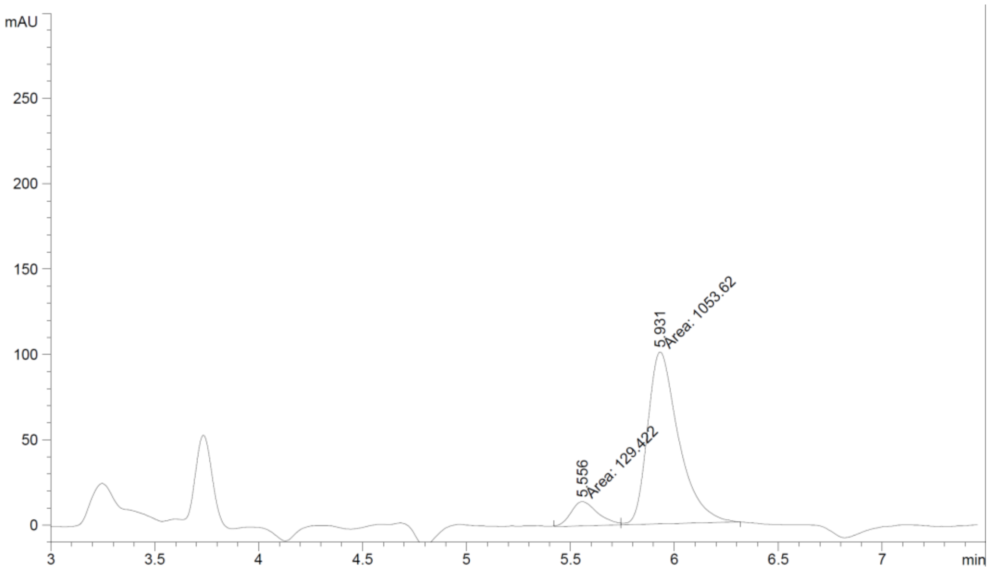

Employing rotaxane 6b as catalyst

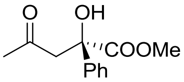

(*R*)-12Me

Peak #	RetTime [min]	Type	Width [min]	Area [mAU*s]	Height [mAU]	Area %
1	5.608	MF	0.1662	40.13816	4.02415	7.7917
2	6.035	FM	0.1882	475.00140	42.07163	92.2083

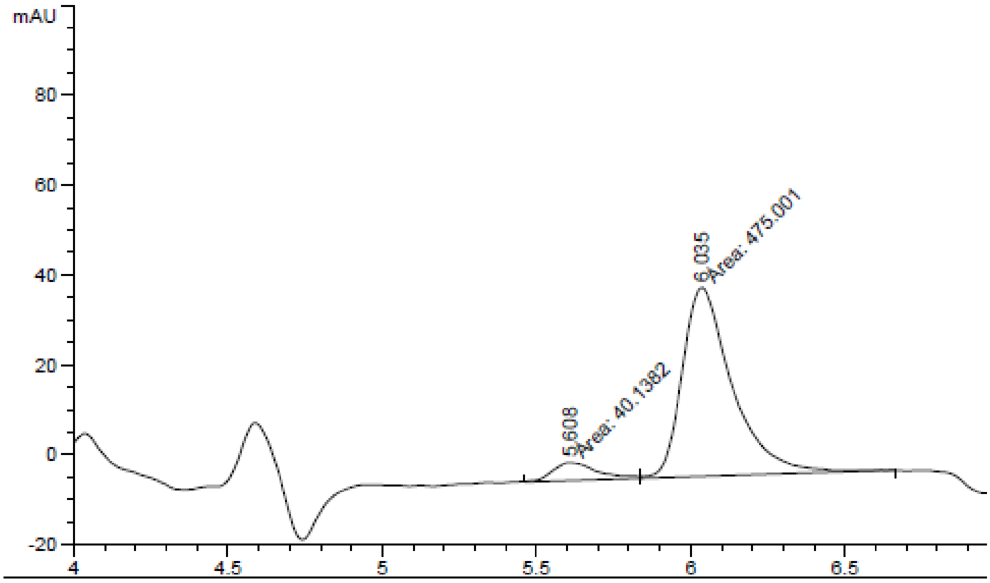

Employing thread 4 as catalyst + 5 equiv. of *N*-hexylthymine

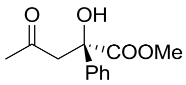

(*R*)-12Me

Peak #	RetTime [min]	Type	Width [min]	Area [mAU*s]	Height [mAU]	Area %
1	5.546	MF	0.1495	482.42035	53.76867	39.8423
2	5.925	FM	0.1764	728.40411	68.80480	60.1577

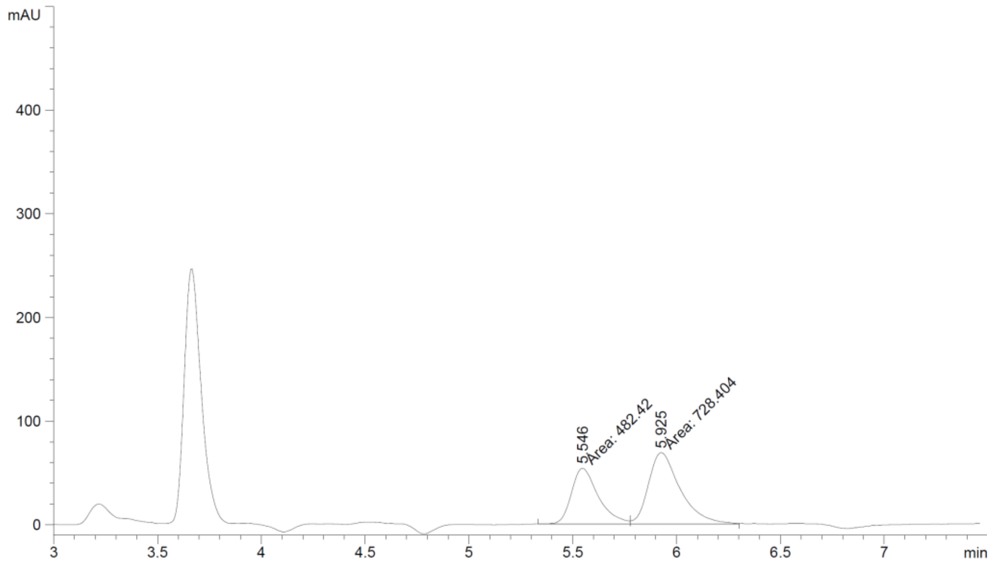

Employing rotaxane 6b as catalyst + 5 equiv. of *N*-hexylthymine

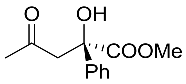

(*R*)-12Me

Peak #	RetTime [min]	Type	Width [min]	Area [mAU*s]	Height [mAU]	Area %
1	5.576	MF	0.1448	191.44476	22.02998	28.0120
2	5.953	FM	0.1793	491.99362	45.73296	71.9880

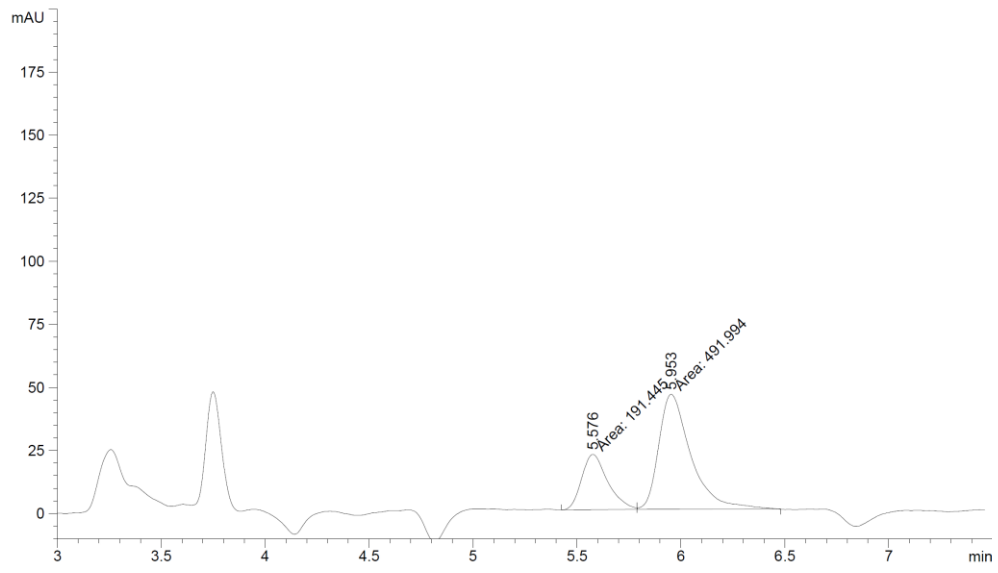
mAU
175
150
125
100
75
50
25
0
3
3.5
4
4.5
5
5.5
6
6.5
7
min
5.576
Area: 191.445
5.953
Area: 491.994
